# Direct All-Atom Nonadiabatic Semiclassical Simulations for Electronic Absorption Spectroscopy of Organic Photovoltaic Non-Fullerene Acceptor in Solution

**DOI:** 10.1021/acs.jpclett.5c00714

**Published:** 2025-04-25

**Authors:** Zengkui Liu, Xiang Sun

**Affiliations:** † Division of Arts and Sciences, NYU Shanghai, 567 West Yangsi Road, Shanghai 200124, China; ‡ NYU-ECNU Center for Computational Chemistry at NYU Shanghai, 3663 Zhongshan Road North, Shanghai 200062, China; ¶ Shanghai Frontiers Science Center of Artificial Intelligence and Deep Learning, NYU Shanghai, 567 West Yangsi Road, Shanghai 200124, China; § Department of Chemistry, New York University, New York, New York 10003, United States; ∥ State Key Laboratory of Precision Spectroscopy, East China Normal University, Shanghai 200062, China

## Abstract

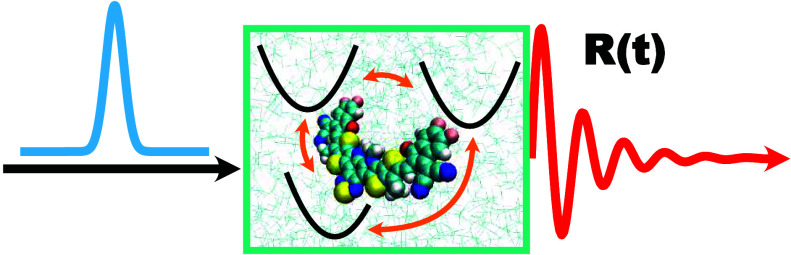

We investigate the linear absorption spectra of the organic photovoltaic nonfullerene acceptor Y6 in chloroform using perturbative and nonperturbative approaches with atomistic details. Direct nonadiabatic semiclassical mapping dynamics reveal population and coherence evolution during and after ultrafast light pulse, revealing dominant absorption to the *S*_1_ state and subsequent oscillatory polarization. The simulated spectra accurately reproduce experimental peak positions and broadening, corresponding to transitions from the ground state to the *S*_1_, *S*_2_, and *S*_6_ excited states. Time-dependent radial distribution functions offer atomistic insights into solvent reorganization in response to charge redistribution. These findings enhance the understanding of nonadiabatic dynamics in Y6 and provide a consistent protocol for simulating electronic spectroscopy in condensed-phase systems.

Electronic spectroscopy is a fundamental tool for characterizing molecular properties pertinent to photochemistry, photosynthesis, and solar energy conversion.^1−6^ Over the past few years, significant theoretical efforts have been dedicated to computing electronic spectra, aiming to interpret experimental results^7−11^ and develop novel spectroscopic methods that provide deeper physical insights.^12−17^ Simulating electronic spectra for disordered condensed matter, such as liquid solutions, presents challenges due to the complexities of light-matter interactions, environmental effects, and nonadiabatic dynamics involved in spectroscopic measurements.^18−28^ In electronic absorption spectroscopy, incident light induces coherence between the ground state and an electronically excited state, probably involving vibrational excitation and leading to vibronic features in the measured spectra. Accurately capturing chromophore-environment interactions, including solute–solvent rearrangements and complex hydrogen bonding—which can be highly anharmonic and exhibit many-body characteristics—is essential. Therefore, faithful simulation of the spectroscopic process is critical for determining the absorption line shape.

One widely adopted approach for calculating the absorption spectra of chromophores involves optimizing the molecular geometry of the chromophore and employing time-dependent density functional theory (TD-DFT) combined with a polarizable continuum model (PCM) to account for implicit solvent effects.^29^ This method yields excitation energies corresponding to the peak positions in the spectrum. However, since single-point calculations do not inherently include vibronic effects, the resulting discrete spectral sticks are often broadened using arbitrary parameters. This arbitrary broadening may not accurately capture finite temperature effects or the fine structure arising from vibronic coupling.

Incorporating realistic broadening into absorption spectra calculations can be achieved through ensemble averaging of molecular conformations sampled from the ground-state potential energy surface (PES) with explicit solvent, utilizing methods such as molecular dynamics (MD)^12,30^ or quantum mechanics/molecular mechanics (QM/MM)^31−34^ at finite temperatures. This approach addresses inhomogeneous and temperature-dependent thermal broadening by sampling the static nuclear density distribution of molecular structures within their environment;^35−37^ however, it does not account for dynamical or vibronic effects. Conversely, the Franck–Condon approach^38,39^ considers the vibronic effects of the chromophore molecule, typically within an implicit solvent framework. This method relies on calculating Franck–Condon overlaps between vibrational states of the ground and electronically excited states, necessitating optimized geometries for both states, corresponding normal modes, and the electronic-vibrational coupling. While this approach effectively captures vibronic structures, it can be computationally intensive for large systems and often omits explicit environmental interactions. Recent advancements have demonstrated that combining nuclear ensemble averaging with the Franck–Condon method yields improved predictions of electronic spectra than the ensemble approach.^32^

The cumulant method^40,41^ offers an alternative approach to modeling spectral line shapes by incorporating dynamical effects besides the vibronic and environmental effects that are captured by the above-mentioned static methods. This technique formulates the linear response function in terms of cumulant expansion that is typically truncated after the second-order cumulant. It effectively assumes Gaussian distribution for the energy gap between the ground and excited states, thereby invoking harmonic approximation. In other words, if a system is accurately described by shifted harmonic potentials of identical curvature, then the Gaussian distribution of energy gap and hence the second order cumulant expansion would be exact, but for realistic systems, the second order cumulant method is an approximation. The second-order cumulant can be written as a function of the spectral density, which is the Fourier transform of the time correlation function (TCF) of the energy gap between two electronic states. This energy-gap TCF could be obtained with all-atom MD simulations with explicit solvent, for instance, which encompasses the system-specific dynamical information with solute–solvent interactions. Optionally, the continuous spectral density can be further discretized to parametrize a Brownian oscillator model (BOM),^40^ which features displaced harmonic oscillators on two states. It should be distinguished between the anharmonic PES effects from sampling and dynamical perspectives. Sampling on anharmonic PES is widely adopted when obtaining energy-gap TCFs,^32,42^ but it does not fully capture the anharmonic effect in the dynamics of spectroscopic response. It is noted that whether or not to construct BOM explicitly, using spectral density in the second-order cumulant approach invokes the harmonic approximation to the effective PES that will be used subsequently for dynamics, which is known analytically. Despite that the spectral density may be derived from atomistic input, the mapped BOM or equivalently using the spectral density loses the atomistic details when performing the dynamical calculation for spectroscopy. Thus, a main drawback of the second-order cumulant approach is that it cannot describe *dynamical* effects on anharmonic PES, which may be important for complex condensed phase systems and some nonlinear spectroscopic features that will vanish for purely harmonic systems.

In this work, we present a direct dynamical protocol of simulating the linear electronic spectroscopy of molecules in liquid solution with atomistic details, which has a consistent theoretical treatment for several aspects, including anharmonic PES, environmental effect, vibronic effect, finite temperature effect, as well as nonadiabatic dynamics that could allow population transfer and coherences between multiple electronically excited states,^43^ plus a realistic simulation of the field-matter interaction in both the perturbative and the nonperturbative approaches.^18,19^ Additionally, various nonadiabatic semiclassical mapping dynamics are tested in the spectroscopic simulations. In both perturbative and nonperturbative treatments, the required TCFs involving electronic coherences are naturally defined. To ensure consistency in treating these coherences, we include the mean-field Ehrenfest dynamics, while the fewest switches surface hopping is not considered, as defining such coherence-related TCFs within that framework is not straightforward.

Figure 1(a) emphasizes that the current method lies at the intersection of all-atom multistate anharmonic Hamiltonian, nonadiabatic dynamics, and perturbative and nonperturbative spectroscopic observables. In particular, we will investigate the linear spectroscopy of an organic photovoltaic (OPV) nonfullerene acceptor (NFA) Y6^44−48^ dissolved in chloroform (trichloromethane, TCM) solution.^49^ As a representative novel NFA, Y6 blended with polymer donors such as PM6 exhibits power conversion efficiency as high as 19.3%.^50^ Additionally, the Y6 molecule has an Acceptor–Donor–Acceptor (A-D-A) complex structure (Figure 1(b)), which may exhibit intramolecular charge transfer (CT) upon photoexcitation in the solution phase (Figure 1(c)), and the most significant excited states including *S*_1_, *S*_2_, and *S*_6_ are shown in Figure 1(d). It would be interesting to see the nonadiabatic dynamics under the direct influence of the explicit external ultrafast light pulse as in the spectroscopic experiment.^49^

**Figure 1:**
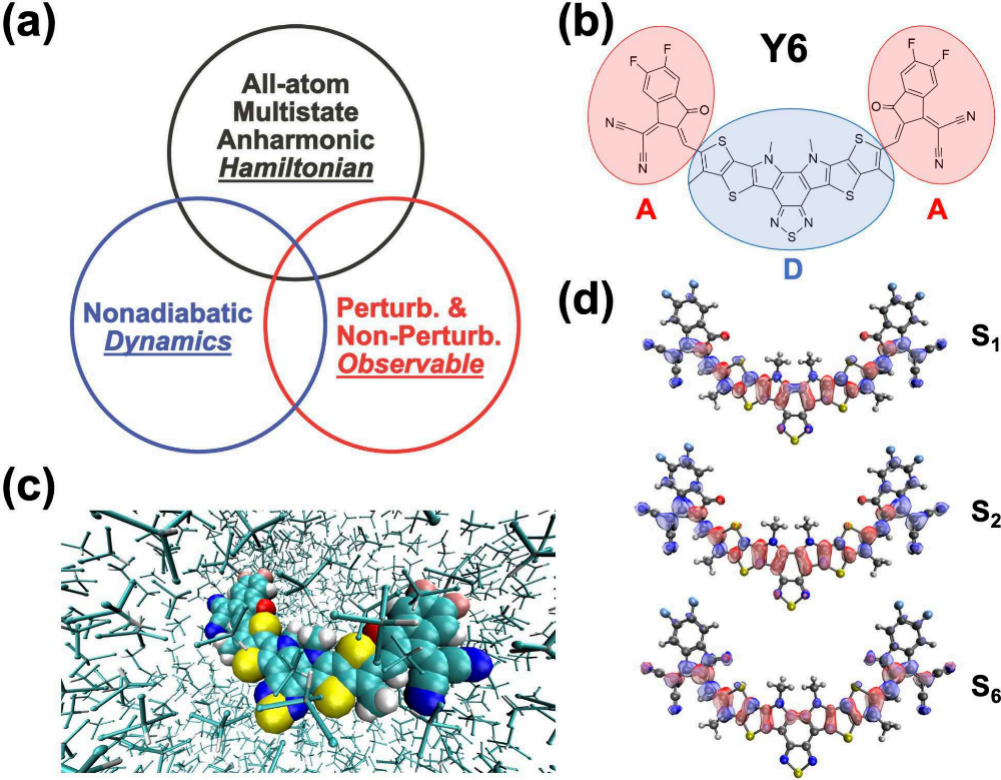
Methodology features and system of interest: (a) the three aspects in spectroscopic simulation: Hamiltonian, dynamics, and spectroscopic observable; (b) the A-D-A molecular structure of organic photovoltaic nonfullerene acceptor Y6; (c) Y6 dissolved in explicit chloroform solution; (d) the detachment–attachment plot of *S*_1_, *S*_2_, and *S*_6_ excited states of Y6.

We employ both perturbative and nonperturbative approaches for simulating the linear absorption spectra.^18,19,40,41,51^ We start by expressing the total Hamiltonian as the sum of *F*-state material Hamiltonian *Ĥ*_*M*_ and the time-dependent light-matter interaction *Ĥ*_int_(*t*) as follows
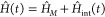
1
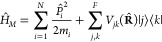
2
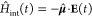
3Here, in the material Hamiltonian, **M** = {*m*_*i*_ |*i* = 1, ···, *N*}, **P̂** = {*P̂*_*i*_ |*i* = 1,..., *N*} and **R̂** = {**R̂**_*i*_ |*i* = 1,..., *N*} are masses, momenta and positions for *N* nuclear DOF, correspondingly, *V*_*jj*_(**R̂**)≡ *V*_*j*_(**R̂**) is the PES of the *j*-th electronic state, *V*_*jk*_(**R̂**) (*j*≠ *k*) is the electronic coupling between the *j*-th and the *k*-th states. Additionally, in the interaction part, the dipole moment operator
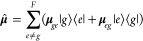
4where **μ**_*eg*_ (*e* ≠ *g*) is the transition dipole moment between the *e*-th excited and the ground (*g*) states, and **E**(*t*) = **ê***E*(*t*) cos(*ω t*–**k**·**r**) is the time-dependent external electric field with the polarization unit vector **ê**, pulse envelope *E*(*t*), leading frequency ω, and wave vector **k**.

First, the perturbative approach is based on the time-dependent perturbation theory where the light-matter interaction is assumed to be weak, and it formulates the absorption line shape *I*(ω) in terms of the linear optical response function *R*(*t*):^40^
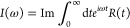
5
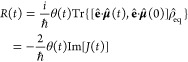
6Here, θ(*t*) is the Heaviside function and Tr(·) is the trace over both electronic and nuclear DOF and the TCF of the dipole moment operator along the electric field is given by
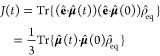
7where the equilibrium density matrix is ρ̂_eq_ = ρ̂_*g*_ |*g*⟩⟨*g*| and the ground state nuclear density is ρ̂_*g*_ = *e*^–*βĤ*_*g*_^/Tr_*N*_{*e*^^–^*βĤ*_*g*_^}. The second line of eq 7 was obtained by applying rotational average for an isotropic liquid system, and **μ̂** = (μ̂_*x*_, μ̂_*y*_, μ̂_*z*_) is the transition dipole of the chromophore in 3-dimensional space and each component μ̂_*a*_ (*a* = *x*, *y*,*z*) is an electronic operator. Within the Condon approximation where the transition dipole moment is assumed constant in the molecular frame, one can simplify the TCF *J*(*t*) as a linear combination of coherence-to-coherence TCFs:^19,40^
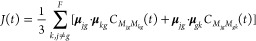
8Here, using the quantum evolution operator  with time-ordered exponential, we express the TCF of the elementary electronic operators *M*_*jk*_ = |*j*⟩ ⟨*k*| and *M*_*mn*_ = |*m*⟩⟨*n*| as
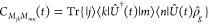
9

Second, the nonperturbative approach^19,51^ does not assume weak light-matter interaction and simulates the realistic nonadiabatic process starting from population of the ground state |*g*⟩⟨*g*| followed by explicit light-matter interaction *Ĥ*_int_(*t*) that induces the electronic coherence between the ground state and the excited states, i.e. |*g*⟩⟨*e*| and |*e*⟩⟨*g*|. The light pulse of short duration τ creates a nonequilibrium state of the system that will undergo the free-induction decay (FID) back to equilibrium, whose dynamics is governed by the field-free Hamiltonian *Ĥ*_*M*_. In this case, the absorption spectrum measures the time evolution of the polarization. The polarization at time *t* after the laser pulse is written as the expectation value of the dipole moment operator projected to the detection polarization direction:
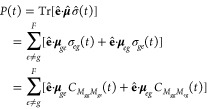
10where the reduced density matrix (RDM) is obtained by performing partial trace for density matrix over the nuclear DOF, namely σ̂(*t*) = Tr_*N*_ [ρ̂(*t*)]and the population-to-coherence TCF *C*_*M*_*gg*_ *M*_*ge*__ (*t*) and *C*_*M*_*gg*_ *M*_*eg*__ (*t*) correspond to starting from a ground-state population and observe a coherence at time *t*, in contrast to the coherence-to-coherence TCF *C*_*M*_*jg*_*M*_*kg*__ (*t*) as used in the perturbative approach. The line shape function in the nonperturbative approach is given by
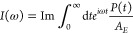
11where *A*_*E*_ = ∫_0_^τ^d*tE*(*t*) is the total electric field exposure for duration τ.

We consider the most important three excited states of the Y6 molecule, i.e., *S*_1_, *S*_2_, and *S*_6_ (Figure 1(d)), which have large oscillator strengths from the ground state, *S*_0_. Among these states, *S*_2_ shows the most significant CT character. In the framework of nonadiabatic mapping dynamics that we employ in this study, the Meyer-Miller-Stock-Thoss Hamiltonian^52,53^ for Y6 dissolved in chloroform with time-dependent light-matter interaction can be represented as below
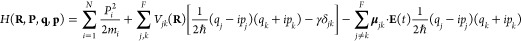
12where the number of electronic states *F* = 4, the electronic couplings *V*_*jk*_ (*j*≠ *k*) are assumed constants under Condon approximation, **q** = {*q*_*i*_ |*i* = 1, ···, *F*} and **p** = {*p*_*i*_ |*i* = 1, ···, *F*} are the position and momentum mapping variables, respectively, γ is the zero-temperature-energy (ZPE) parameter (default γ = 1/2 if not mentioned otherwise in this work), and the transition dipole moments **μ**_*jk*_ only exist between the ground state and the excited states. Detailed information on the electronic states of Y6 can be found in Supporting Information.

Once the initial conditions for (**R**,**P**, **q**,**p**) are known, the nonadiabatic propagation of the mapping Hamiltonian reduces to solving its corresponding Hamilton’s equations.^42^ In this sense, the equations of motion for the nonadiabatic propagation is the same for the linearized semiclassical (LSC),^54−59^ symmetrical quasiclassical (SQC),^60,61^ classical mapping model (CMM),^62−64^ spin mapping model (SPM),^65,66^ as well as the mixed quantum-classical mean-field (MF) Ehrenfest dynamics^67^ (γ = 0). These nonadiabatic mapping dynamical methods differ in the initial sampling of the mapping variables, observable evaluation, and ZPE choice.

In particular, TCF *C*_*M*_*jk*_*M*_*mn*__(*t*) evaluated in the mapping dynamics is written as
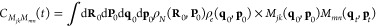
13Here, ρ_*N*_(**R**_0_,**P**_0_) is the initial nuclear phase space distribution such as the equilibrated ground state, ρ_*e*_(**q**_0_,**p**_0_) is the initial distribution of the electronic mapping variables, for example, in LSC methods
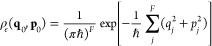
14and in CMM method, mapping variables are sampled on a hypersphere such that the total electronic population is unity:
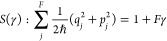
15For the observable evaluation in eq 13, the time *t* observable in LSC1 (or Poisson bracket mapping equation)^58^ and LSC2 (or LSC-initial value representation)^56^ correspond to
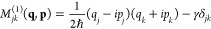
16
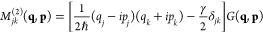
17respectively, where γ = 1/2 and . The time-0 observable in both LSC1 and LSC2 will be the same as *M*_*jk*_^(2)^ (**q**_0_, **p**_0_) in eq 17 with *G*(**q**_0_, **p**_0_) used for the electronic initial sampling . The resolution of identity (RI) for the electronic DOF could improve the estimation of population,^54,59^ and combining the RI trick with the LSC approaches yields resolution-of-identity linearized semiclassical 1–3 (RI-LSC1–3) methods (see Supporting Information for details).

Additionally, in CMM method, the time-0 and time-*t* observables are given by
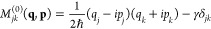
18
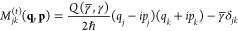
19respectively, where  and . The Q, P, and W schemes in the SPM method correspond to choosing , respectively. It is noted that the uniform sampling across all electronic states employed in the LSC and CMM methods makes them suitable for obtaining the multiple coherence-to-coherence and population-to-coherence TCFs with the same batch of nonadiabatic dynamical trajectories in the perturbative approach. This is in contrast with the methods such as Ehrenfest and SQC methods, where the initial sampling of the electronic mapping variables are focused on a single state, thus requiring multiple batches of nonadiabatic simulation as in the perturbative approach. The nonperturbative approach, however, would only require to start from the equilibrated ground state, so it could combine with the Ehrenfest and SQC more straightforwardly than the perturbative approach. If not mentioned otherwise, the time step in all dynamical calculations is chosen to be *δt* = 0.1 fs. In the nonperturbative approach, the applied square laser pulse of strength *E* = 2.57 × 10^10^ V/m lasts for 0.1 fs. For more details of other dynamical methods such as Ehrenfest and SQC methods, refer to ref (43) and the Supporting Information.

Figure 2 shows the time-domain response function *R*(*t*) as in the perturbative approach and the polarization *P*(*t*) as in the nonperturbative approach of the Y6 chloroform solution. With the amplitude of both quantities normalized, the time profiles of both *R*(*t*) and *P*(*t*) are rather similar, which indicates the validity of the perturbative treatment. Their similarity could be traced back to the fact that the external light pulse used in the direct nonperturbative nonadiabatic simulation is rather narrow (one time step), so the polarization that is the convolution of the response function and the narrow external electric field is close to the material-only response function. The slightly augmented oscillation in polarization than the response function at time *t* > 40 fs is probably due to the finite width of the pulse as opposed to a true δ-function pulse shape. It is observed that the response functions obtained with different nonadiabatic dynamical methods are similar and so are the polarizations. The Y6 solution at room temperature is probably represented by the BOM that falls in the friendly parameter space where different mixed quantum-classical and semiclassical nonadiabatic dynamics generate agreeing results, which was discussed in a recent benchmark work.^68^ Even if there were some slight differences in the time-domain results across different dynamical methods, they might be easier to be observed in the frequency domain.

**Figure 2:**
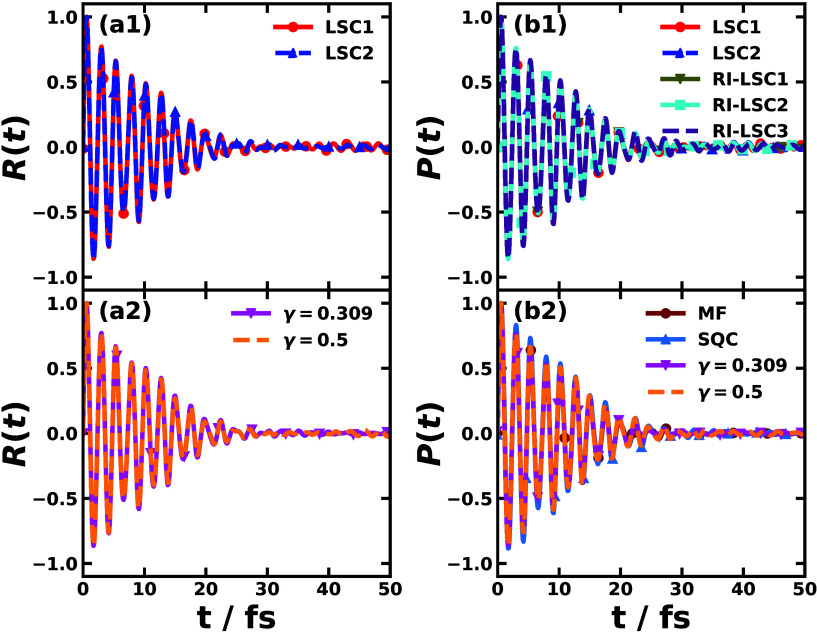
Time-domain response function of Y6 dissolved in chloroform calculated with the perturbative approach (left panels) via nonadiabatic dynamics including LSC1 and LSC2 (a1), and CMM with ZPE parameters γ = 0.309 (SPM-W) and γ = 0.5 (a2); the time-dependent polarization after the external laser pulse of strength *E* = 2.57 × 10^10^ V/m and duration 0.1 fs calculated with the nonperturbative approach (right panels) via nonadiabatic dynamics including LSC1, LSC2, and RI-LSC1–3 (b1) and MF, SQC, and CMM with ZPE parameters γ = 0.309 (SPM-W) and γ = 0.5 (b2). The results are normalized according to their maximal amplitudes.

The main result of the paper is Figure 3, which depicts the frequency-domain linear absorption spectra of the Y6 chloroform solution, as obtained by Fourier transforming the response function and the polarization in the perturbative (eq 5) and nonperturbative approaches (eq 11), respectively. The main spectroscopic features of Y6 solution from the simulation agree well with the experimental measurement^49^ in terms of peaks at about 1.7, 2.1, and 2.7 eV corresponding to *S*_0_ to *S*_1_, *S*_2_, and *S*_6_ absorption, respectively. The main peak at 1.7 eV from the simulation is seen to reproduce the line shape broadening compared with the experiment, except for the shoulder around 1.9 eV. The shoulder might have originated from the Y6 dimer formation, which was not explicitly treated in this work. Also, the experimental peaks at 2.1 and 2.7 eV have a higher baseline than the simulation result. The spectra calculated with the perturbative and nonperturbative approaches are rather similar and the nonadiabatic dynamical methods including LSC1, LSC2, RI-LSC1–3, CMM (γ = 0.309, or SPM-W), CMM (γ = 0.5), and Ehrenfest lead to the same spectra. The only exception is SQC method in the nonperturbative approach, which shows a negative peak at 2.3 eV and the reason is that the underlying binning algorithm leads to an unphysical oscillatory *S*_1_ population. Additionally, the nonadiabatic effects can be tested by turning off the electronic coupling between excited states but still having the vibronic coupling between electronic and nuclear DOF. The simulation without nonadiabatic effects yields a lower *S*_2_ peak and a higher *S*_6_ peak, which is more distinct from the experimental results than the original simulated spectra with nonadiabatic effects. This highlights the importance of the nonadiabatic effects in calculating the absorption spectroscopy.

**Figure 3:**
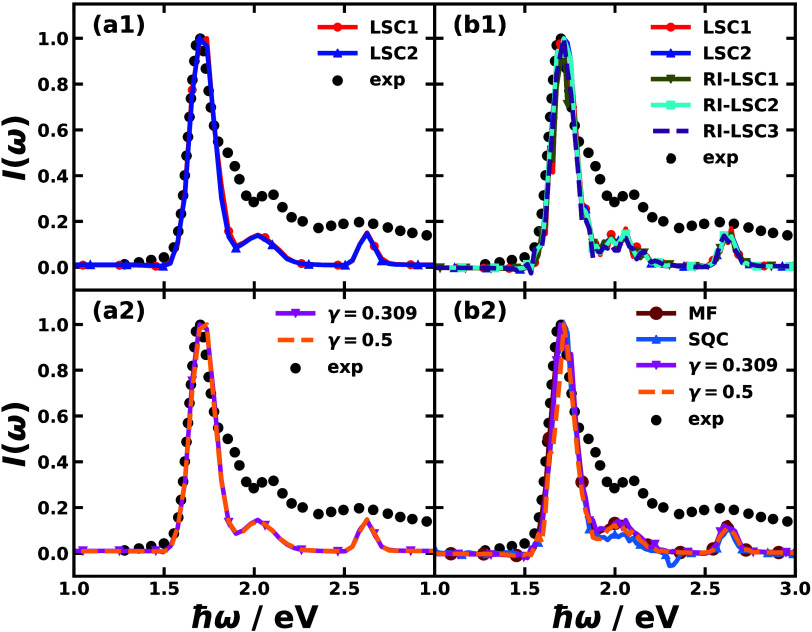
Linear absorption spectra of Y6 dissolved in chloroform solution obtained via the perturbative approach (left panels) and nonperturbative approach (right panels) using various nonadiabatic dynamics including LSC1, LSC2, RI-LSC1–3, MF, SQC, and CMM with γ = 0.309 (SPM-W) and CMM with γ = 0.5, in comparison with the experimental spectra (black circles) adapted from ref (49). The spectral line shapes are normalized according to their maximal amplitudes, but the peak positions are not shifted.

We also test the perturbative approach on coumarin 153 (C153)’s benzene solution at 300 K (refer to the Supporting Information for details). The ground *S*_0_ state and the first excited *S*_1_ state of C153 are considered here. The response function and the linear spectra are shown in Figures S5 and S6 in the Supporting Information. The simulated spectra reproduce the peak position of the experimental result and about 60% peak width for the absorption of *S*_0_→*S*_1_ transition. Additionally, the simulated spectra of the C153 solution obtained with various semiclassical dynamics methods are very similar, also seen in the Y6 case. Overall, we believe the theoretical spectra capture the main experimental features remarkably, especially considering that the theoretical spectra are obtained directly from the nonadiabatic dynamical simulation without frequency-shifting or artificial broadening.

Besides the spectra, we can also obtain detailed nonadiabatic dynamics from the direct simulation of the nonperturbative approach. For example, the population dynamics during and after the external light pulse are plotted in Figure 4. The system was prepared to be in equilibrium with the ground state, and during the light pulse (see Figure 4(a)), there is a rapid population transfer from the ground state to the three excited states, and the transition to *S*_1_ state is the most significant to about 0.55. After the light-matter interaction period, the population transfer dramatically slows down, which relies completely on the electronic couplings that are smaller than the light-matter interaction. In the long time scale, however, the populations of *S*_2_ and *S*_6_ exhibit complementary oscillatory dynamics until reaching the plateau after 30 fs, as shown in Figure 4(b). The similar population dynamics obtained with other nonadiabatic dynamical methods are shown in Figure S1 in the Supporting Information. Moreover, the charge transfer amount dynamics of Y6 after the photoexcitation as depicted in Figure S2 in the Supporting Information is seen to reflect the population oscillation between *S*_2_ and *S*_6_ states.

**Figure 4:**
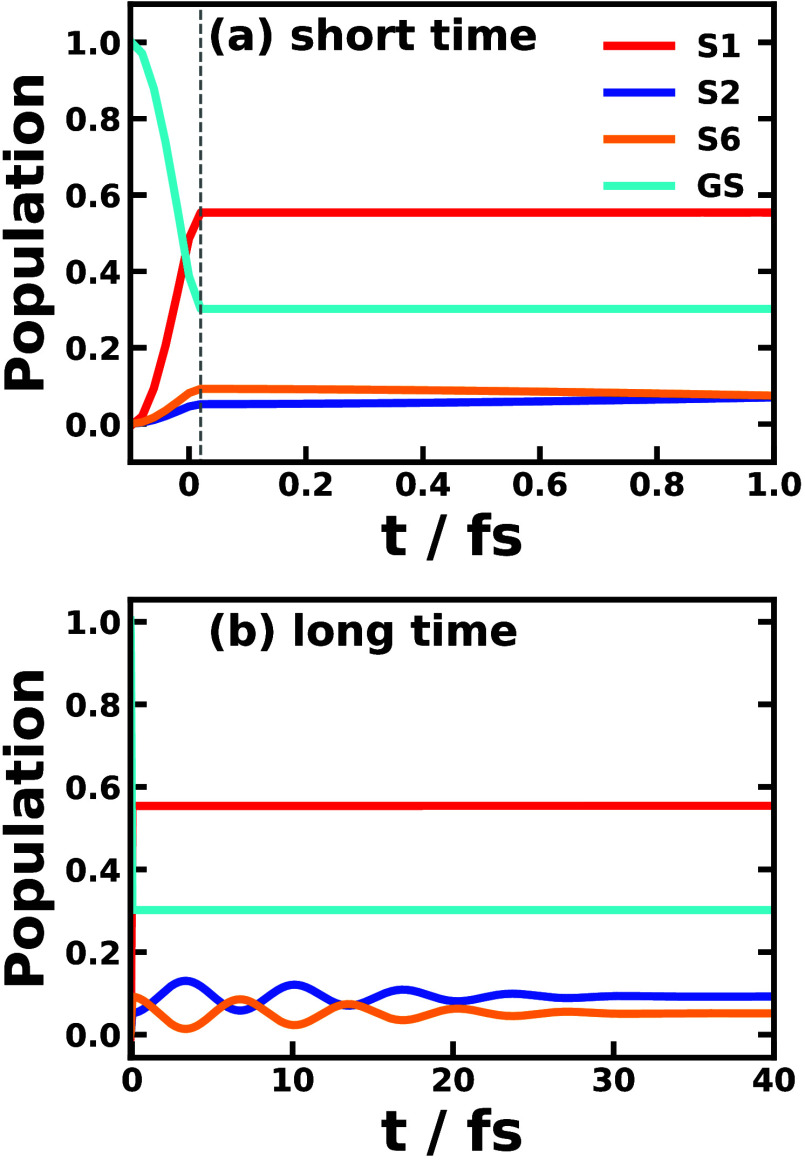
Population transfer dynamics of Y6 dissolved in chloroform via RI-LSC2 method with time step *δ t* = 0.02 fs during the light pulse (duration 0.1 fs) (left to the dashed line in (a)) and after the light-matter interaction (right to the dashed line in (a)) in the short time (a) and long time (b) scales simulated in the nonperturbative approach. A total of four electronic states are involved in the nonadiabatic dynamics, including the excited *S*_1_, *S*_2_, *S*_6_ states, and the ground state (GS).

Moreover, we can also obtain the entire RDM dynamics from the direct nonadiabatic dynamical simulations in the nonperturbative approach. Figure 5 shows the RDM dynamics obtained with RI-LSC2, CMM (γ = 0.309), and SQC methods, where the upper and lower triangle panels are the real and imaginary parts of the coherences, respectively. It is seen that the coherence between the ground state and the first excited state, i.e., σ_14_(*t*), contributes the most to the polarization in Figure 2(b). This is expected since the *S*_0_→*S*_1_ transition is the main electronic transition for the absorption spectra, thus a large coherence between the two states. In contrast, the coherences between other excited states (*S*_2_ and *S*_6_) and the ground state are small in amplitude, which slightly modulates the polarization. Furthermore, the coherences between *S*_1_ and *S*_2_/*S*_6_ show a larger amplitude than the coherence between *S*_2_ and *S*_6_, which could be attributed to the relatively large population of *S*_1_ state about 0.75. We note that the *S*_1_ population in Figure 5 about 0.75 is larger than in Figure 4 about 0.55, and the reason is that the time affected by the external laser pulse (i.e., the pulse duration plus one time step) is longer in Figure 5, hence more population transfer to *S*_1_ would occur. As shown in Figure 5, RI-LSC2 and CMM (γ = 0.309) produce almost the same RDM dynamics. However, the SQC generates more oscillatory *S*_1_ population and weaker coherences than the other methods [see Figure 5 and Figure S1(g)]. It might result from the trajectories that fall out of the population window functions in SQC simulation^60^ and in turn, give rise to the negative peak in the absorption line shape in Figure 3.

**Figure 5:**
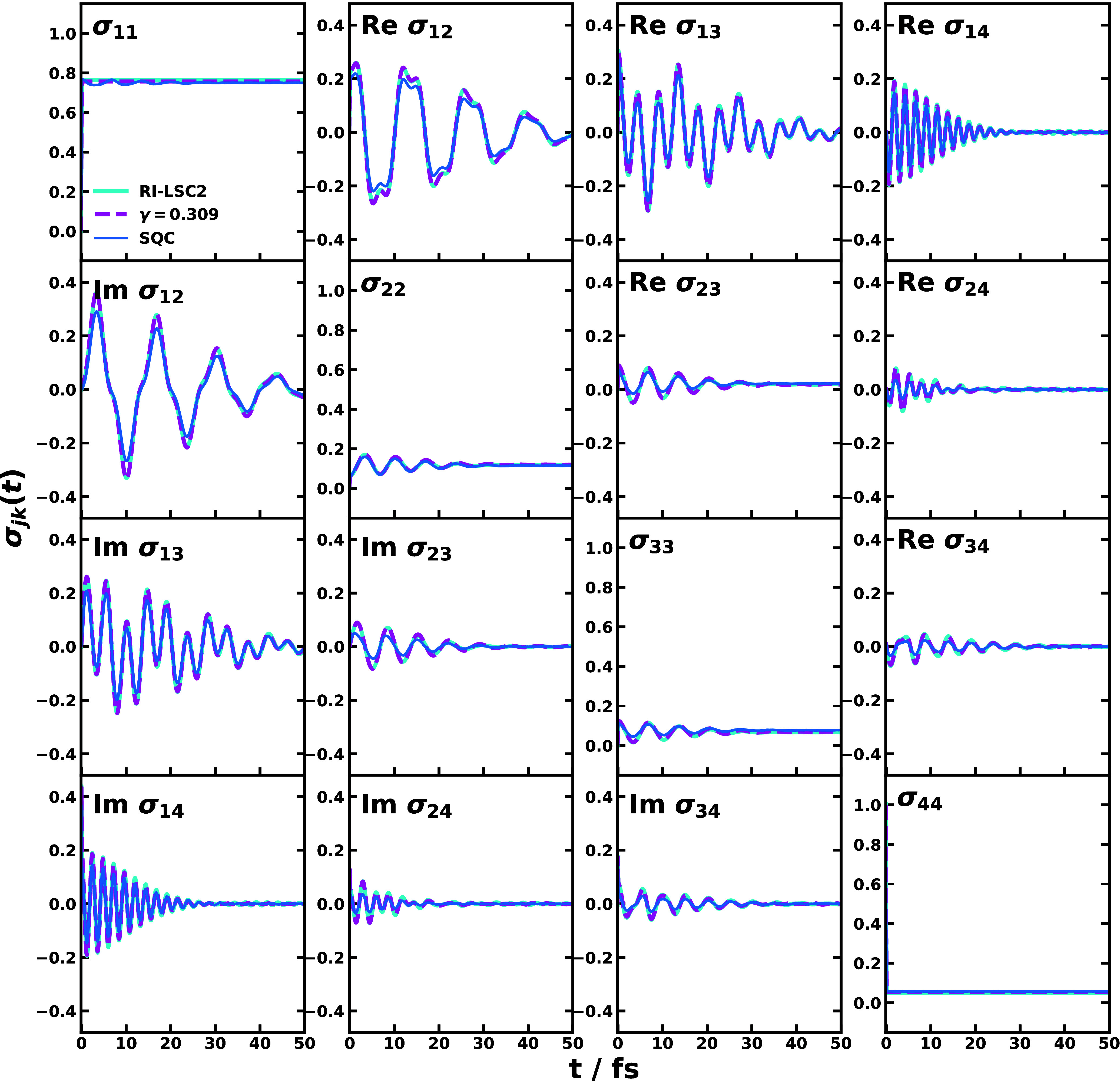
Reduced density matrix dynamics of Y6 dissolved in chloroform obtained with RI-LSC2, CMM (γ = 0.309, SPM-W), and SQC methods with time step *δt* = 0.1 fs after the external light pulse (duration 0.1 fs) in the nonperturbative approach. The state labels 1–4 correspond to the excited *S*_1_, *S*_2_, *S*_6_, and the ground states, respectively.

Figure 6 shows atomistic information involved in the nonadiabatic dynamics in the direct nonperturbative approach — the radial distribution function (RDF) change for a specific atom in the solvent with respect to the surface of the donor moiety of Y6 molecule. The first row in Figure 6 is the equilibrated ground state RDF for the hydrogen atom and the chlorine atom with respect to the donor moiety of Y6, i.e., the time-0 structural property. The second to the fourth rows of Figure 6 show the RDF change up to 0.1 and 0.4 ps when simulated with Ehrenfest, LSC, and CMM (γ = 0.309) dynamical methods, respectively. After the explicit light-matter interaction, the hydrogen of chloroform with respect to the donor moiety of Y6 (Figure 6(a)) is seen decreased by 10% at around 0.25 nm, which is driven by the populated excited states with CT character and the donor moiety gets more positively charged, thereby repelling the hydrogen atom in chloroform. The opposite trend is observed in the RDF changes between the chlorine atom in solvent and the donor of Y6 (Figure 6(b)), where the more positively charged donor moiety of Y6 attracts the negatively charged chlorine atom in chloroform from 0.5 nm region to 0.4 nm region. The opposite RDF trend can be observed between the solvent and the acceptor moieties of Y6 as shown in Figures S3 and S4 in the Supporting Information.

**Figure 6:**
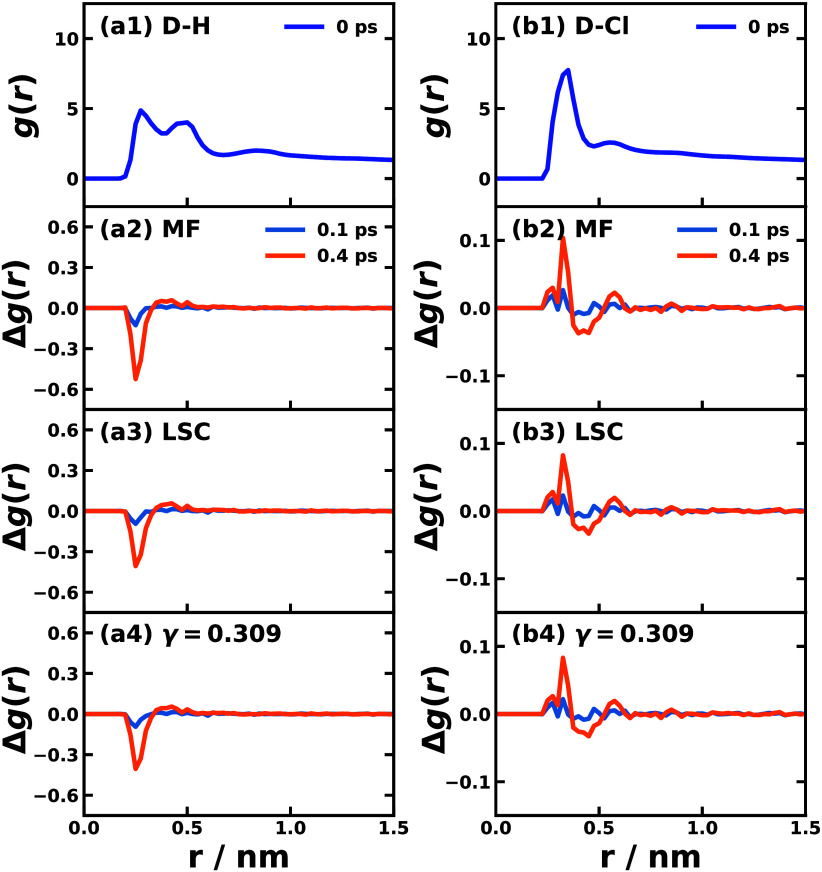
Radial distribution function (RDF) of the hydrogen (left) and chlorine (right) atoms of chloroform with respect to the surface of donor moiety of Y6. The first row (a1,b1) corresponds to the RDF of the equilibrium ground state, the rows 2 to 4 correspond to the RDF changes up to 0.1 ps (blue) and 0.4 ps (orange) after the applied light pulse in nonperturbative approach via MF (a2,b2), LSC (a3,b3), and CMM with γ = 0.309 (SPM-W) (a4,b4).

We have systematically investigated the linear absorption spectra of Y6 in chloroform using perturbative and nonperturbative methodologies, providing a detailed analysis of nonadiabatic population and coherence dynamics. These computational approaches account for the vibronic effect, environmental effect, and anharmonic realistic interactions in a consistent manner within direct nonadiabatic dynamics for liquid solutions and yield an agreement with the experimental spectra. The direct dynamical simulation of the nonperturbative approach enabled us to resolve the transient population transfer under light excitation and subsequent electronic transition dynamics. Our study reveals that the LSC, MF, SPM, and CMM methods yield consistent results, while the SQC method exhibits small discrepancies in absorption features. However, high temperature and small reorganization energy alone are not sufficient to guarantee agreement among various nonadiabatic dynamics methods. A benchmark comparison with exact results using reduced effective models—such as the multistate harmonic (MSH) model^42^—is ultimately necessary, especially when all-atom quantum dynamics are not feasible. Additionally, solvent structural reorganization following photoexcitation has been demonstrated with time-dependent RDF, reflecting CT-induced solvent redistribution around the donor and acceptor moieties of Y6. These findings contribute to the broader understanding of excitonic and solvent-mediated effects in organic photovoltaic systems and pave the way for refined theoretical approaches in modeling electronic spectroscopy in complex molecular environments using a consistent treatment for atomistic multistate Hamiltonian, nonadiabatic dynamics, and spectroscopic observables.

*Simulation Summary*. The excitation energies, transition dipoles from the ground state to excited states, diabatic couplings, and atomic charges were calculated with time-dependent density functional theory (TDDFT) on the level of ω*B97X-D/6–31G(d,p) using polarizable continuum model of ε_0_ = 3.0 and the tuned range separation parameter is ω = 0.11 using Q-Chem 6.0. The excited states (*S*_1_, *S*_2_, *S*_6_) were selected with the minimal oscillator strength threshold of 0.2 and maximum excitation energy threshold of 3.0 eV. The all-atom PESs are based on the generalized Amber force field, where the atomic charges and the excitation energies correspond to the TDDFT calculation for the ground state and the three excited states. The initial nuclear positions and momenta are sampled from equilibrated ground state Y6 with 1632 explicit chloroform solvent molecules in box 60.9 × 60.9 × 60.9 Å^3^ with periodic boundary conditions at 300 K. The nonadiabatic dynamics simulations of the Y6 molecule dissolved in the explicit solvent environment for the linear absorption spectra are performed using a nuclear time step of 0.1 fs and an electronic time step of 0.005 fs by averaging over 2 × 10^4^ trajectories in the perturbative approach and 10^5^ trajectories in the nonperturbative approach. For more details of the simulation, refer to the Supporting Information.

## Supporting Information Available

Details of the nonadiabatic semiclassical mapping dynamics, quantum chemistry calculation of Y6, and all-atom simulations, population dynamics obtained with all nonadiabatic dynamics, charge transfer amount dynamics, and radial distribution function change between solvent and acceptor moieties of Y6, simulation details of quantum chemistry calculation and nonadiabatic dynamics for C153 in benzene solution at 300 K, and the response function and linear absorption spectroscopy (PDF)
Transparent Peer Review report available (PDF)
