# Supplementary material for: Direct All-Atom Nonadiabatic Semiclassical Simulations for Electronic Absorption Spectroscopy of Organic Photovoltaic Non-Fullerene Acceptor in Solution
Source: J Phys Chem Lett. 2025 Apr 25;16(18):4463–73. doi: 10.1021/acs.jpclett.5c00714 (PMC12067435; doi:10.1021/acs.jpclett.5c00714)
Supplement: Supplementary file 1 — jz5c00714_si_001.pdf [file jz5c00714_si_001.pdf]

Supporting Information:
Direct All-Atom Nonadiabatic Semiclassical Simulations for Electronic Absorption Spectroscopy of Organic Photovoltaic Non-Fullerene Acceptor in Solution

Zengkui Liu[1,2,3,4] and Xiang Sun[1,3,2,5,4,a)]
[1)]Division of Arts and Sciences, NYU Shanghai, 567 West Yangsi Road, Shanghai 200124, China
[2)]NYU-ECNU Center for Computational Chemistry at NYU Shanghai, 3663 Zhongshan Road North, Shanghai 200062, China
[3)]Shanghai Frontiers Science Center of Artificial Intelligence and Deep Learning, NYU Shanghai, 567 West Yangsi Road, Shanghai, 200124, China
[4)]Department of Chemistry, New York University, New York, New York 10003, United States
[5)]State Key Laboratory of Precision Spectroscopy, East China Normal University, Shanghai 200062, China

I. NONADIABATIC SEMICLASSICAL MAPPING DYNAMICS

The detailed description of atomistic nonadiabatic semiclassical mapping dynamics can be found in Ref. 1. Here, we briefly describe the nonadiabatic dynamical calculation for the spectroscopy simulation. We start with the general F-state Hamiltonian with external laser field $\mathbf{E}(t)$:

$$\hat{H}(t) = \sum_{i=1}^{N} \frac{\hat{P}_i^2}{2m_i} + \sum_{j,k}^{F} \left(V_{jk}(\hat{\mathbf{R}}) - \boldsymbol{\mu}_{jk} \cdot \mathbf{E}(t) \right) |j\rangle\langle k|, \tag{S1}$$

where $\hat{\mathbf{R}} = \{\hat{R}_j | j = 1, \cdots, N\}$ and $\hat{\mathbf{P}} = \{\hat{P}_j | j = 1, \cdots, N\}$ are position and momentum operators for N nuclear degrees of freedom (DOF), accordingly; m_i $(i = 1, \ldots, N)$ is the mass of each nuclear DOF; $\hat{V} = \sum_{jk} V_{jk}(\hat{\mathbf{R}})|j\rangle\langle k|$ is the potential energy term in the diabatic basis, $\hat{V}_{jj}$ is the potential energy surface of the j-th electronic state, $\hat{V}_{jk}$ $(j \neq k)$ is the electronic coupling between the j-th state and the k-th state; $\boldsymbol{\mu}_{eg}$ $(e \neq g)$ is the transition dipole moment between the e-th excited and the ground (g) states; $\mathbf{E}(t) = \hat{\mathbf{e}}E(t)\cos(\omega t - \mathbf{k}\cdot\mathbf{r})$ is the time-dependent external electric field with the polarization unit vector $\hat{\mathbf{e}}$, pulse envelope $E(t)$, leading frequency ω, and wave vector $\mathbf{k}$. Here, we invoke the long-wavelength approximation, which assumes the molecular size is much smaller than the wavelength of the incident field, thus the electric field at different positions and $\mathbf{k}\cdot\mathbf{r}$ can be treated as constants.

Within nonadiabatic mapping dynamics, the Meyer-Miller-Stock-Thoss (MMST) Hamiltonian[2,3] is adopted and given by

$$H(\mathbf{R}, \mathbf{P}, \mathbf{q}, \mathbf{p}) = \sum_{i=1}^{N} \frac{\hat{P}_i^2}{2m_i} + \sum_{j,k}^{F} \left(V_{jk}(\mathbf{R}) - \boldsymbol{\mu}_{jk} \cdot \mathbf{E}(t) \right) \left[\frac{1}{2} (q_j - ip_j)(q_k + ip_k) - \gamma \delta_{jk} \right], \tag{S2}$$

where $\mathbf{p} = \{q_j | j = 1, \cdots, F\}$ and $\mathbf{q} = \{q_j | j = 1, \cdots, F\}$ are the phase space mapping variables, γ is the zero-point-energy (ZPE) parameter, and our default value is $\gamma = 0.5$ if not stated otherwise.

The electronic reduced density matrix (RDM) is defined as

$$\hat{\sigma}(t) = \mathrm{Tr}_N[\hat{\rho}(t)] = \sum_{j,k}^{F} \sigma_{jk}(t)|j\rangle\langle k|, \tag{S3}$$

where $\hat{\rho}(t)$ is the overall density operator and $\mathrm{Tr}_{N/e}[\cdot]$ denotes the trace over the nuclear and electronic DOF, respectively. The initial density operator is assumed to be separable with respect to nuclear and electronic DOF, namely $\hat{\rho}(0) = \hat{\rho}_N(0) \otimes \hat{\sigma}(0) = \sum_{j,k}^{F} \hat{\rho}_N(0) \otimes \sigma_{jk}(0)|j\rangle\langle k|$, where $\hat{\rho}_N(0)$ is the initial nuclear density operator, and in the current study equilibrium density of the ground state $\hat{\rho}_g = e^{-\beta \hat{H}_g} / \mathrm{Tr}_N[e^{-\beta \hat{H}_g}]$ is used. The RDM at time t is given by

$$\sigma_{kj}(t) = \sum_{m,n}^{F} \sigma_{mn}(0) \mathrm{Tr}_{N,e} \left[\hat{\rho}(0)|m\rangle\langle n|\hat{U}^{\dagger}(t)|j\rangle\langle k|\hat{U}(t) \right] = \sum_{m,n}^{F} \sigma_{mn}(0) C_{M_{mn}M_{jk}}(t), \tag{S4}$$

[a)]Electronic mail: xiang.sun@nyu.edu

where $M_{jk} = |j\rangle\langle k|$ and the elementary electronic operator TCF is defined as

$$C_{M_{mn}M_{jk}}(t) = \mathrm{Tr}_{N,e}\left\{|m\rangle\langle n|\hat{U}^\dagger(t)|j\rangle\langle k|\hat{U}(t)\hat{\rho}_g\right\}. \tag{S5}$$

Here, the evolution operator is $\hat{U}(t) = \exp_+\left[-\frac{i}{\hbar}\int_0^t \mathrm{d}t'\hat{H}(t')\right]$ with time-ordered exponential function.

Linearized semiclassical mapping methods. The linearized semiclassical (LSC) mapping dynamics formulates a quantum time correlation function in the classical Wigner form

$$C_{AB}(t) = \mathrm{Tr}_{N,e}\left[\hat{\rho}\hat{A}\hat{U}^\dagger(t)\hat{B}\hat{U}(t)\right] \tag{S6}$$

$$= \frac{1}{(2\pi\hbar)^{(N+F)}}\int \mathrm{d}\mathbf{R}_0\mathrm{d}\mathbf{P}_0\mathrm{d}\mathbf{q}_0\mathrm{d}\mathbf{p}_0\left[\rho_N(0)\right]_\mathrm{W}(\mathbf{R}_0,\mathbf{P}_0)\left[A\right]_\mathrm{W}(\mathbf{q}_0,\mathbf{p}_0)\left[B\right]_\mathrm{W}(\mathbf{q}_t,\mathbf{p}_t), \tag{S7}$$

where $\mathbf{R}_0, \mathbf{P}_0$ denote the initial nuclear position and momenta, $\mathbf{q}_0, \mathbf{p}_0$ and $\mathbf{q}_t, \mathbf{p}_t$ denote the electronic mapping variables at time 0 and at time t, respectively, and $[O]_\mathrm{W}(\mathbf{q},\mathbf{p})$ denotes the Wigner-transformation for electronic operator $\hat{O}$:

$$[O]_\mathrm{W} = \int \mathrm{d}\mathbf{z}e^{-i\mathbf{z}\cdot\mathbf{p}/\hbar}\left\langle \mathbf{q}+\frac{\mathbf{z}}{2}\middle|\hat{O}(\hat{\mathbf{q}},\hat{\mathbf{p}})\middle|\mathbf{q}-\frac{\mathbf{z}}{2}\right\rangle. \tag{S8}$$

There are two ways to treat semiclassical mapping relations, which are referred to as LSC mapping #1 and #2, respectively:[4]

$$\left[M_{jk}\right]_\mathrm{W}^{(1)}(\mathbf{q},\mathbf{p}) = \frac{1}{2\hbar}(q_j - ip_j)(q_k + ip_k) - \gamma\delta_{jk}, \tag{S9}$$

$$\left[M_{jk}\right]_\mathrm{W}^{(2)}(\mathbf{q},\mathbf{p}) = \left[\frac{1}{2\hbar}(q_j - ip_j)(q_k + ip_k) - \frac{\gamma}{2}\delta_{jk}\right]G(\mathbf{q},\mathbf{p}), \tag{S10}$$

where $\gamma = 1/2$ and $G(\mathbf{q},\mathbf{p}) = 2^{F+2}\exp\left[-\frac{1}{\hbar}(\mathbf{p}^\mathrm{T}\mathbf{p}+\mathbf{q}^\mathrm{T}\mathbf{q})\right]$, which is used for initial sampling of mapping variables in LSC mapping dynamics. The nuclear initial sampling is done according to the Wigner function

$$\left[\rho_N(0)\right]_\mathrm{W}(\mathbf{R},\mathbf{P}) = \int \mathrm{d}\mathbf{Z}e^{-i\mathbf{Z}\cdot\mathbf{P}/\hbar}\left\langle \mathbf{R}+\frac{\mathbf{Z}}{2}\middle|\hat{\rho}_N(0)\middle|\mathbf{R}-\frac{\mathbf{Z}}{2}\right\rangle, \tag{S11}$$

which can be approximated with the classical nuclear distribution. Thus, in the LSC1 and LSC2 methods, the RDM is given by

$$\sigma_{jk}^{\mathrm{LSC1/2}} = \left(\frac{1}{2\pi\hbar}\right)^{N+F}\sum_{n,m}^{F}\sigma_{mn}(0)\int \mathrm{d}\mathbf{R}_0\mathrm{d}\mathbf{P}_0\mathrm{d}\mathbf{q}_0\mathrm{d}\mathbf{p}_0\left[\rho_N(0)\right]_\mathrm{W}(\mathbf{R}_0,\mathbf{P}_0)$$
$$\times\left[M_{mn}\right]_\mathrm{W}^{(2)}(\mathbf{q}_0,\mathbf{p}_0)\left[M_{kj}\right]_\mathrm{W}^{(1)/(2)}(\mathbf{q}_t,\mathbf{p}_t). \tag{S12}$$

Additionally, the resolution of identity (RI) trick has been shown to improve the population estimation.[5,6] In this case, the population and coherence observables are formulated in terms of TCF of identity operator $\hat{I}$, traceless operator $\hat{Q}_j = F\hat{M}_{jj} - \sum_j \hat{M}_{jj}$, and the elementary coherence M_{jk} $(j \neq k)$ if the initial electronic state is $\hat{\sigma}(0) = |m\rangle\langle m|$:[4,7,8]

$$\sigma_{jj}(t) = \frac{1}{F^2}\left[F + C_{\hat{I}\hat{Q}_j}(t) + C_{\hat{Q}_m\hat{Q}_j}(t)\right], \tag{S13}$$

$$\sigma_{jk}(t) = \frac{1}{F}\left[C_{\hat{I}\hat{M}_{kj}}(t) + C_{\hat{Q}_m\hat{M}_{kj}}(t)\right]. \tag{S14}$$

The traceless operator in LSC mapping No. 1 and No.2 are given by

$$[Q_j]_\mathrm{W}^{(1)/(2)}(\mathbf{q},\mathbf{p}) = F\,[M_{jj}]_\mathrm{W}^{(1)/(2)}(\mathbf{q},\mathbf{p}) - \sum_k^F [M_{kk}]_\mathrm{W}^{(1)/(2)}(\mathbf{q},\mathbf{p}). \tag{S15}$$

The three RI-modified LSC approaches uses mapping No. 2 for time-t observables, but they differ by:

1. RI-LSC1 maps the identity operator as 1 and uses mapping No. 1 for $\hat{Q}_m$.
2. RI-LSC2 maps the identity operator as 1 and uses mapping No. 2 for $\hat{Q}_m$.
3. RI-LSC3 maps the identity operator as $G(\mathbf{q},\mathbf{p})$ and uses mapping No. 2 for $\hat{Q}_m$.

Classical mapping model and spin-mapping model. The extended classical mapping model (CMM)[9–12] require the initial sampling of the mapping variables in a constraint hypersurface such that the total electronic population is unity:

$$S(\gamma):\sum_{j=1}^{F}\left[\frac{1}{2\hbar}\left(q_j^2+p_j^2\right)-\gamma\right]=1. \tag{S16}$$

The TCF between initial RDM element $|m\rangle\langle n|$ and final RDM element $|j\rangle\langle k|$ (applies to both population and coherence) is given by

$$\begin{aligned}C_{M_{mn}M_{jk}}(t)=&\frac{1}{(2\pi\hbar)^N}\int \mathrm{d}\mathbf{R}_0\mathrm{d}\mathbf{P}_0\,[\rho_N(0)]_{\mathrm{W}}(\mathbf{R}_0,\mathbf{P}_0)\\&\times\int_{S(\gamma)}\mathrm{d}\mathbf{q}_0\mathrm{d}\mathbf{p}_0\left[\frac{1}{2\hbar}(q_m(0)-ip_m(0))(q_n(0)+ip_n(0))-\gamma\delta_{nm}\right]\\&\times\left[\frac{Q(\overline{\gamma},\gamma)}{2\hbar}(q_j(t)-ip_j(t))(q_k(t)+ip_k(t))-\overline{\gamma}\delta_{jk}\right],\end{aligned} \tag{S17}$$

where $\overline{\gamma}=\frac{1-\gamma}{1+F\gamma}$ and $Q(\overline{\gamma},\gamma)=\frac{1+F\overline{\gamma}}{1+F\gamma}$. The Q, P, and W schemes in the spin-mapping model (SPM)[13,14] correspond to choosing ZPE parameter $\gamma=0,1,\frac{\sqrt{F+1}-1}{F}$, respectively.

Symmetrical quasiclassical method. The symmetrical quasiclassical (SQC) method[15–17] shares the MMST Hamiltonian and the equations of motion as the above-mentioned methods, but SQC has a unique way to determine population by windowing the action mapping variables. The action-angle mapping variables $\mathbf{n}=\{n_j|j=1,\cdots,F\}$ and $\mathbf{u}=\{u_j|j=1,\cdots,F\}$ can be related to $(\mathbf{q},\mathbf{p})$ by $q_j=\sqrt{2(n_j+\gamma)\hbar}\cos(u_j)$ and $p_j=-\sqrt{2(n_j-\gamma)}\sin(u_j)$. The SQC with triangle window was implemented with recommended ZPE parameter $\gamma=1/3$, and the triangle window functions are given by

$$\begin{aligned}[M_{jj}]_W^{\text{(SQC-tri)}}(\mathbf{n},\mathbf{u})&=w_1(n_j)\prod_{l\neq j}^{F}w_0(n_j,n_l),\\ \left[M_{kj}\right]_W^{\text{(SQC-tri)}}(\mathbf{n},\mathbf{u})&=e^{i(u_j-u_k)}w_{\frac{1}{2}}(n_k)w_{\frac{1}{2}}(n_j)\prod_{l\neq j,k}^{F}w_0(n_{j,k},n_l),\end{aligned} \tag{S18}$$

where $w_a(n_j)=(2-\gamma-n_j)^{2-F}$ when $-\gamma<n-a<1-\gamma$ and $w_a=0$ otherwise; $w_0(n_j,n_l)=1$ when $n_l<2-2\gamma-n_j$ and $w_0=0$ otherwise. In practice, the RDM elements should be normalized via $\sigma_{jk}(t)=\sigma_{jk}^{\mathrm{raw}}(t)/\sum_l^F\sigma_{ll}^{\mathrm{raw}}(t)$, where $\sigma^{\mathrm{raw}}(t)$ is the raw electronic reduced density matrix at time t, and this means that some of the trajectories will fall out of all the population windows. The initial value of mapping variables $\mathbf{n}$ are uniformly distributed within the initial window and the $\mathbf{u}$ is uniformly distribution in $[0,2\pi)$.

Mean-field Ehrenfest dynamics. The mean-field (MF) Ehrenfest dynamics[18,19] was usually considered as mixed quantum-classical dynamics. However, if we express the wavefunction expansion coefficients as in $|\Psi(t)\rangle=\sum_j c_j(t)|\psi_j\rangle$

$$c_j=\frac{1}{\sqrt{2\hbar}}(q_j+ip_j) \tag{S19}$$

the RDM element would be equivalent to the case when ZPE vanishes ($\gamma=0$), i.e.,

$$\sigma_{jk}=c_jc_k^*=\frac{1}{2\hbar}(q_k+ip_j)(q_k-ip_k). \tag{S20}$$

From the quantum Liouville equation $\frac{\partial}{\partial t}\hat{\sigma}(t)=-\frac{i}{\hbar}\left[\hat{V}(\mathbf{R}_t),\,\hat{\sigma}(t)\right]$, the time evolution of the RDM is

$$\hat{\sigma}(t+\Delta t)=\hat{U}(t+\Delta t,t)\hat{\sigma}(t)\hat{U}^{\dagger}(t+\Delta t,t). \tag{S21}$$

The nuclear equations of motion are given by $\dot{\mathbf{R}}=\mathbf{P}$, $\dot{\mathbf{P}}=-\frac{\partial}{\partial\mathbf{R}}V_{\mathrm{mf}}(\mathbf{R})=\mathbf{F}_{\mathrm{mf}}(\mathbf{R})$, where the mean-field potential is

$$V_{\mathrm{mf}}(\mathbf{R}_t)=\mathrm{Tr}\left[\hat{\sigma}(\hat{V}-\hat{\boldsymbol{\mu}}\cdot\mathbf{E}(t))\right]=\sum_{j,k}^{F}\sigma_{jk}(t)\left[V_{kj}(\mathbf{R}_t)-\boldsymbol{\mu}_{kj}\cdot\mathbf{E}(t)\right]. \tag{S22}$$

Ehrenfest dynamics has no initial sampling for the electronic DOF since all the initial electronic RDM elements $\sigma_{jk}(0)$ are known. The initially occupied electronic state is prepared by setting $c_{\mathrm{occ}}=1$.

II. QUANTUM CHEMISTRY CALCULATION OF Y6

The geometry of the molecule Y6 is adopted from Ref. 20. The excited state properties, like excitation energies, Merz-Kollman (MK) restrained electrostatic potential (RESP) atomic partial charges, and fragment charge difference (FCD) coupling, are calculated using time-dependent density functional theory (TDDFT) with Q-Chem 6.0 at the level of ω*B97X-D/6-31G(d,p) with an SSVPE polarizable continuum model (PCM) whose dielectric constant is 3.0 and tuned range-separation parameter of $\omega = 0.11$.[21–24] The RESP partial atomic charges of a chloroform solvent are calculated using Gaussian16[25] at the level of CCSD/aug-cc-pVDZ. Excited states of interest are selected with the minimal oscillator strength threshold of 0.2 and maximum excitation energy threshold of 3 eV. The selected bright excited states including S_1, S_2, and S_6 are included in the nonadiabatic dynamical simulation together with the ground state. We neglected the dark states in the nonadiabatic dynamical simulation since they are not expected to be heavily populated in the first tens of fs after the photoexcitation. For completeness, the excitation energies of the dark excited states S_3, S_4, and S_5 are 2.40, 2.46, and 2.50 eV, respectively and the oscillator strengths are 0.09, 0.02, and 0.03, respectively.

TABLE S1. Energy minima ε_j, energy corrections W_j, oscillator strength ζ_j, and charge transfer amount ΔQ_j of four electronic states, electronic couplings Γ_{jk} and reorganization energies $E_r^{(jk)}$ between different pairs of states in the conformations with the minimum ground state energy of Y6. Here, electronic states $j,k = 1,2,3,4$ correspond to S1, S2, S6, and ground (g) states, respectively. The electronic couplings between any excited states ($j < 4$) and the ground state are assumed to be zero. The transition dipole between the ground state and the excited states $\boldsymbol{\mu}_{ge} = (\mu_{ge,x}, \mu_{ge,y}, \mu_{ge,z})$ ($e = S1,\ S2,\ S6$) is given in atomic unit. Energy unit is eV except for $E_r^{(jk)}$ in kJ/mol. Charge transfer amount's unit is e.

Properties	Y6
$\varepsilon_1(\mathrm{S}_1)$	1.72
$\varepsilon_2(\mathrm{S}_2)$	2.09
$\varepsilon_3(\mathrm{S}_6)$	2.56
$\varepsilon_4(g)$	0
$W_1(\mathrm{S}_1)$	0.8125
$W_2(\mathrm{S}_2)$	1.3584
$W_3(\mathrm{S}_6)$	2.6367
$W_4(g)$	0
$\zeta_1(\mathrm{S}_1)$	2.538
$\zeta_2(\mathrm{S}_2)$	0.274
$\zeta_3(\mathrm{S}_6)$	0.630
$\Delta Q_1(\mathrm{S}_1)$	0.308
$\Delta Q_2(\mathrm{S}_2)$	0.727
$\Delta Q_3(\mathrm{S}_6)$	0.187
Γ_{12}	-2.3×10^{-5}
Γ_{13}	-1.6×10^{-4}
Γ_{23}	-1.8×10^{-1}
Γ_{j4}	0
$E_r^{(12)}$	4.701
$E_r^{(13)}$	3.327
$E_r^{(14)}$	8.004
$E_r^{(23)}$	3.546
$E_r^{(24)}$	20.477
$E_r^{(34)}$	6.811
$\boldsymbol{\mu}_{gS1}$	$(-7.76, 0, 0)$
$\boldsymbol{\mu}_{gS2}$	$(0, 2.31, 0)$
$\boldsymbol{\mu}_{gS6}$	$(0, 3.16, 0)$

III. ALL-ATOM SIMULATION OF Y6

The general AMBER force field is applied to construct the force-field Hamiltonian, where the atomic partial charges are replaced by the state-specific RESP charges calculated in the quantum chemistry section and the state-specific energy corrections in Table S1 are applied to correct the rest parts of energy contribution of the GAFF. The Y6 chloroform solution consists of a Y6 solute and 1,623 chloroform molecules. All-atom molecular dynamics (MD) and nonadiabatic semiclassical dynamics

simulations are performed using QCDyn with OpenMM 7.5. In all MD simulations, a harmonic restraint of 100 kcal/mol/Å^2 is applied to the Y6 molecule. Electrostatic interactions are computed using the particle mesh Ewald (PME) method with a 9 Å cutoff, which also applies to the Van der Waals nonbonding interactions. The SHAKE algorithm is used to constrain all covalent bonds involving hydrogen atoms.

MD preparation was performed on the ground-state potential energy surface (PES). The nuclear time step is chosen to be $\delta t = 1$ fs, unless otherwise specified. Energy minimization is performed with a tolerance of 10 kcal/mol and a 10,000-cycle limit. The simulation box is heated to 300 K over 100 ps using a Langevin thermostat with a collision frequency of 1 ps^{-1}, which is also employed throughout all MD simulations for temperature control. For NPT equilibration, a Monte Carlo barostat maintains system pressure at 1 bar (100 kPa) for 2 ns, with barostat adjustments attempted every 25 steps. The average simulation box size of the Y6 solution is $60.9 \times 60.9 \times 60.9$ Å^3. NVT equilibration follows for 2 ns at 300 K, after which the nuclear DOF are sampled every 1 ps along the NVT trajectory at 300 K.

The force field energies for the excited states of Y6 solution require correction to include the electronic excitation energies. The total potential energy of the j-th excited state is given by

$$\begin{aligned} V_j(\mathbf{R}) =& V_j^{\mathrm{FF}}(\mathbf{R}) - \langle V_j^{\mathrm{FF}}(\mathbf{R}) - V_g^{\mathrm{FF}}(\mathbf{R})\rangle + E_{jg}^{\mathrm{QM}}(\mathbf{r}) \\ \equiv& V_j^{\mathrm{FF}}(\mathbf{R}) + W_j(\mathbf{r}). \end{aligned} \tag{S23}$$

Here, $W_j(\mathbf{r})$ is the energy correction of j-th state for the solute with geometry $\mathbf{r}$ (see Table S1), $V_j^{\mathrm{FF}}(\mathbf{R})$ is the original force-field potential energy of the j-th state, $E_{jg}^{\mathrm{QM}}(\mathbf{r})$ is the gap-phase excitation energy on the j-th state with respect to the ground state calculated by quantum chemistry. Also, the ensemble average over the equilibrium distribution of the ground state is denoted as $\langle \bullet \rangle = \int \mathrm{d}\mathbf{R}\mathrm{d}\mathbf{P} \bullet \exp\{-\beta H_g(\mathbf{R},\mathbf{P})\}$. There is no energy correction required for the reference ground state.

In the subsequent nonadiabatic molecular dynamics (NAMD) simulations, the nuclear propagation time step is chosen as 0.1 fs. All-atom mapping dynamics employ a fourth-order Runge-Kutta (RK4) algorithm to propagate the mapping variables 20 times per nuclear step. The perturbative TCFs are averaged over 2×10^4 NAMD trajectories, with initial nuclear phase points equilibrated on the ground-state PES, and all the possible initial RDM elements.

The non-perturbative approach signals require 1×10^5 trajectories for all the semiclassical or quasiclassical dynamics. In the NAMD simulation, initial nuclear DOF are equilibrated on the ground-state PES, and the initial electronic state is set to the ground state before the field-matter interaction. The rotating wave approximation (RWA) Hamiltonian is used in the non-perturbative approach to compute the linear absorption spectra, with an incident field frequency of 1.72 eV, a Rabi frequency between the S_1 state and the ground state $\hbar\chi_{S_1,g} = 10.5$ eV, and a square pulse envelope of 0.1 fs applied in the simulation.

Under the RWA, we assume the ground-excited transition frequency ω_{ge} is close to the light field frequency ω, so their difference will be surviving term compared with highly oscillatory sum term. Within RWA, operators are transformed as

$$\widetilde{A} = e^{i\hat{H}_{\mathrm{rot}}t/\hbar}\hat{A}e^{-i\hat{H}_{\mathrm{rot}}t/\hbar}, \tag{S24}$$

where

$$\hat{H}_{\mathrm{rot}} = \hbar\omega\sum_{j\neq g}^{F}|j\rangle\langle j| = \hbar\omega(\hat{1} - |g\rangle\langle g|). \tag{S25}$$

Thus, the effective Hamiltonian in RWA $\widetilde{H}_{\mathrm{eff}} = \widetilde{H}_M + \widetilde{H}_{int}$ is the sum of material Hamiltonian and field-matter interaction Hamiltonian:

$$\widetilde{H}_M = \sum_{i=1}^{N}\frac{\hat{P}_i^2}{2m_i} + V_g(\mathbf{R})|g\rangle\langle g| + \sum_{j,k\neq g}^{F}\left[V_{jk}(\mathbf{R}) - \hbar\omega\delta_{jk}\right]|j\rangle\langle k|, \tag{S26}$$

$$\widetilde{H}_{\mathrm{int}}(t) = -\frac{\hbar}{2}\sum_{j\neq g}^{F}\left[\chi_{jg}(t)e^{i\mathbf{k}\cdot\mathbf{r}}|j\rangle\langle g| + \chi_{gj}(t)e^{-i\mathbf{k}\cdot\mathbf{r}}|g\rangle\langle j|\right], \tag{S27}$$

where Rabi frequency is defined as

$$\chi_{jg}(t) = \frac{1}{\hbar}\boldsymbol{\mu}_{jg}\cdot\mathbf{E}(t). \tag{S28}$$

The equation of motion of RDM in the rotating frame becomes

$$\frac{\mathrm{d}}{\mathrm{d}t}\widetilde{\sigma}(t) = -\frac{i}{\hbar}\left[\widetilde{H}_{\mathrm{eff}}, \widetilde{\sigma}(t)\right] \tag{S29}$$

where the RDM in the rotating frame remain the same as the original RDM except for the coherences between the ground state and the excited states:

$$\begin{aligned}\widetilde{\sigma}_{jg} =&\sigma_{jg}e^{i\omega t}\\ \widetilde{\sigma}_{gj} =&\sigma_{gj}e^{-i\omega t}\end{aligned} \qquad (j \neq g). \tag{S30}$$

The time-dependent charge-transferred amount is defined as follows:

$$\Delta Q(t) = \sum_{j \neq g}^{F} \Delta Q_j \sigma_{jj}(t), \tag{S31}$$

where ΔQ_j is the CT amount from the donor moiety of the Y6 to the acceptor moieties on both sides of the Y6 for the j-th excited electronic state (see Table S1).

IV. LINEAR ABSORPTION SPECTRA OF C153 IN BENZENE SOLUTION

The geometry of the gas-phase C153 molecule is optimized at the level of B3LYP/cc-pVDZ, and the excited state properties like the MK RESP atomic partial charges and charges are evaluated using TDDFT calculation at the level of B3LYP/cc-pVTZ with SSVPE PCM for benzene (dielectric constant, $\varepsilon_0 = 2.3$). The gap-phase excitation energies are evaluated using TDDFT at the level of B3LYP/cc-pVTZ. All quantum chemistry calculation of C153 is performed with the Q-Chem of version 6.0.[21] The RESP partial atomic charges of benzene molecule are calculated using Gaussian16[26] at the level of CAM-B3LYP/cc-pVTZ.

One C153 solute and 1463 benzene solvents are packed up for the C153 benzene solution. All molecular dynamics and all-atom semiclassical dynamics simulations are performed using QCDyn with OpenMM 7.5. In all MD simulations of the C153 benzene solution, a harmonic force of 23.9 kcal/mol/Å^2 is applied to restraint the solute C153; PME is utilized to calculate the electrostatic interaction with cutoff radius of 9 Å; the nonbonding interaction cutoff radius is set to be 9 Å and the SHAKE algorithm is utilized to restrain all covalent bonding including H-atom. All MD propagation is on the ground state PES. The nuclear propagation step length is 1 fs as is not explicitly specified. The minimization uses a tolerance of 10 kcal/mol and a maximum cycle limit of 10000. The simulation box is heated to 300 K at a constant temperature-raising rate within 100 ps using the Langevin thermostat with the collision frequency of 1 ps^{-1}. The same Langevin thermostat is utilized for all MD simulations for temperature control. The NPT equilibrium utilized a Monte-Carlo barostat to keep the pressure of the system at 1 bar (100 kPa) for 2 ns and the Monte-Carlo barostat is tried per 25 steps. The averaged box size of the C153 system is $60.8 \times 60.8 \times 60.8$ Å^3. The NVT equilibrium lasts for 2 ns at 300 K, and after the NVT equilibrium, the nuclear DOF's canonical distribution is sampled per 1 ps at 300 K along the NVT trajectory.

The force field energy correction for C153 employs the quantum chemical energy gaps of the gas-phase solute to correct the force field energy gap of the solute

$$\begin{aligned}V_j(\mathbf{R}) =&V_j^{\mathrm{FF}}(\mathbf{R}) - \left(E_j^{\mathrm{FF}}(\mathbf{r}) - E_g^{\mathrm{FF}}(\mathbf{r})\right) + E_{jg}^{\mathrm{QM}}(\mathbf{r})\\ =&V_j^{\mathrm{FF}}(\mathbf{R}) + W_j(\mathbf{r})\end{aligned} \tag{S32}$$

where $W_j(\mathbf{r})$ is the j-th state energy correction of the solute with the geometry $\mathbf{r}$, $V_j^{\mathrm{FF}}(\mathbf{R})$ is the force-field potential energy of the entire solution system on the j-th state, $E_{jg}^{\mathrm{QM}}(\mathbf{r})$ is the gap-phase solute excitation energy of the j-th state with respect to the ground state calculated by quantum chemistry, and $E_j^{\mathrm{FF}}(\mathbf{r})$ is the force-field potential energy of the solute alone on the j-th state. Here, the excitation energy $E_{\mathrm{S_1,g}}^{\mathrm{QM}} = 3.38$ eV and the energy correction is $W_{\mathrm{S_1}} = 3.84$ eV.

The two-state all-atom Hamiltonian is constructed for C153 in benzene solution, involving the ground S_0 and the first excited S_1 state. The transition dipole between the S_0 and S_1 states is $\boldsymbol{\mu}_{S_0,S_1} = (0.83, 0.40, 2.06)$ au, and the diabatic coupling between the two states is zero. The nuclear step length of the all-atom NAMD is 0.1 fs. All-atom mapping dynamics utilized RK4 algorithm to propagate the mapping variables propagated 20 times during one nuclear step. The perturbation theory signals are averaged over 2×10^4 NAMD trajectories whose initial nuclear phase points are equilibrated on the ground state PES and the initial RDM elements use all RDM conditions.

Figure S5 shows the time-domain response functions $R(t)$ in the perturbative approach for C153 in the benzene solution at 300 K. In general, the response functions via different semiclassical dynamics are similar to each other for C153 in benzene at room temperature. Figure S6 provides similar linear absorption spectra $I(\lambda)$ via the perturbation theory approach for C153 benzene solution. The simulated spectra can capture the experimentally measured peak position at 420 nm and the peak width via the perturbative approach can reproduce 60% experimental peak width.

V. SUPPLEMENTAL FIGURES

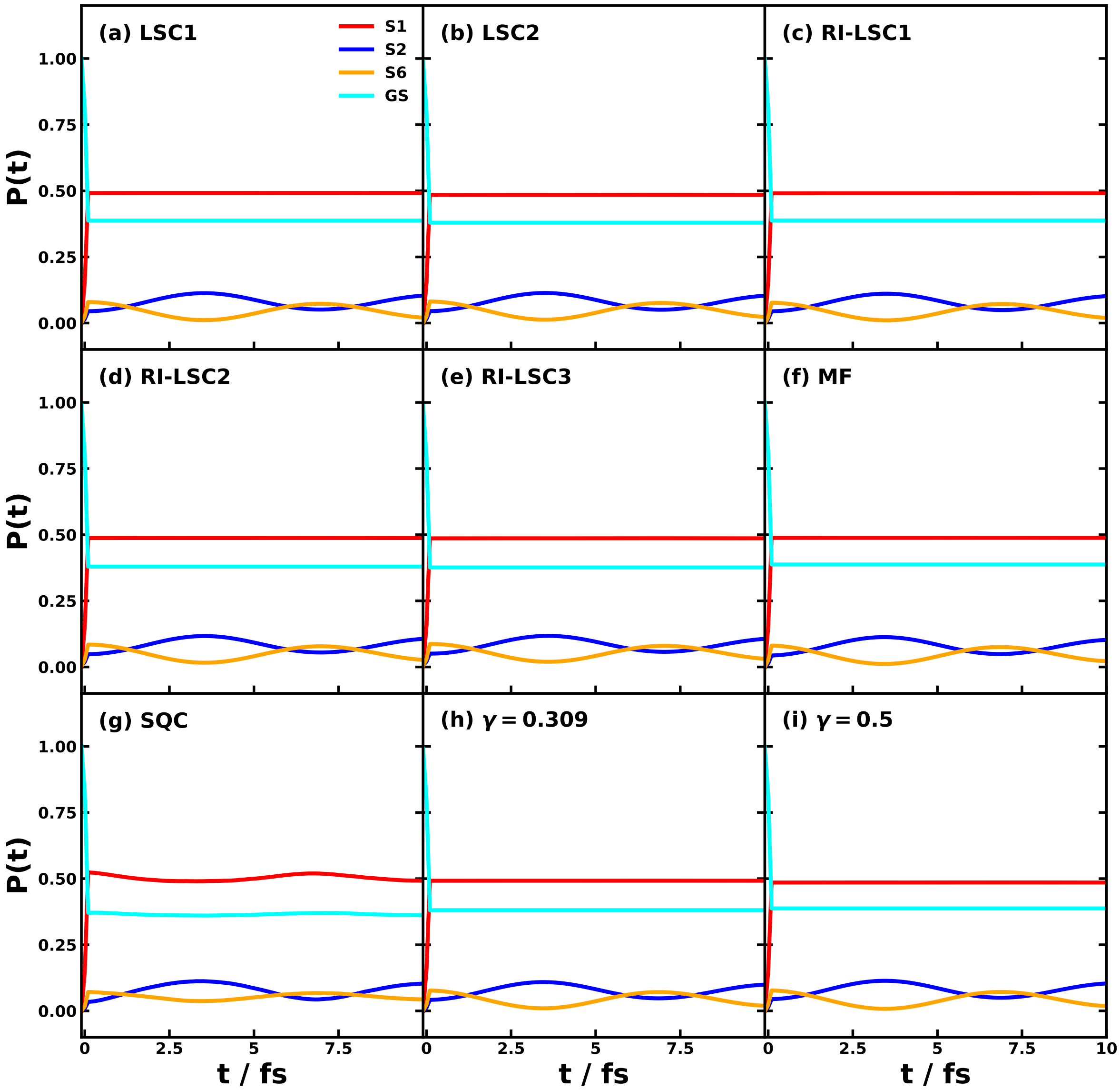

FIG. S1. Population dynamics of Y6's chloroform solution are obtained via the polarization simulation approach by different mapping dynamics shown in panels (a) LSC1, (b) LSC2, (c) RI-LSC1, (d) RI-LSC2, (e) RI-LSC3, (f) MF, (g) SQC, (h) CMM ($\gamma = 0.5$) and (i) SPM-W/CMM with $\gamma = 0.309$. The spectral results are normalized to make their maxima equal to 1.

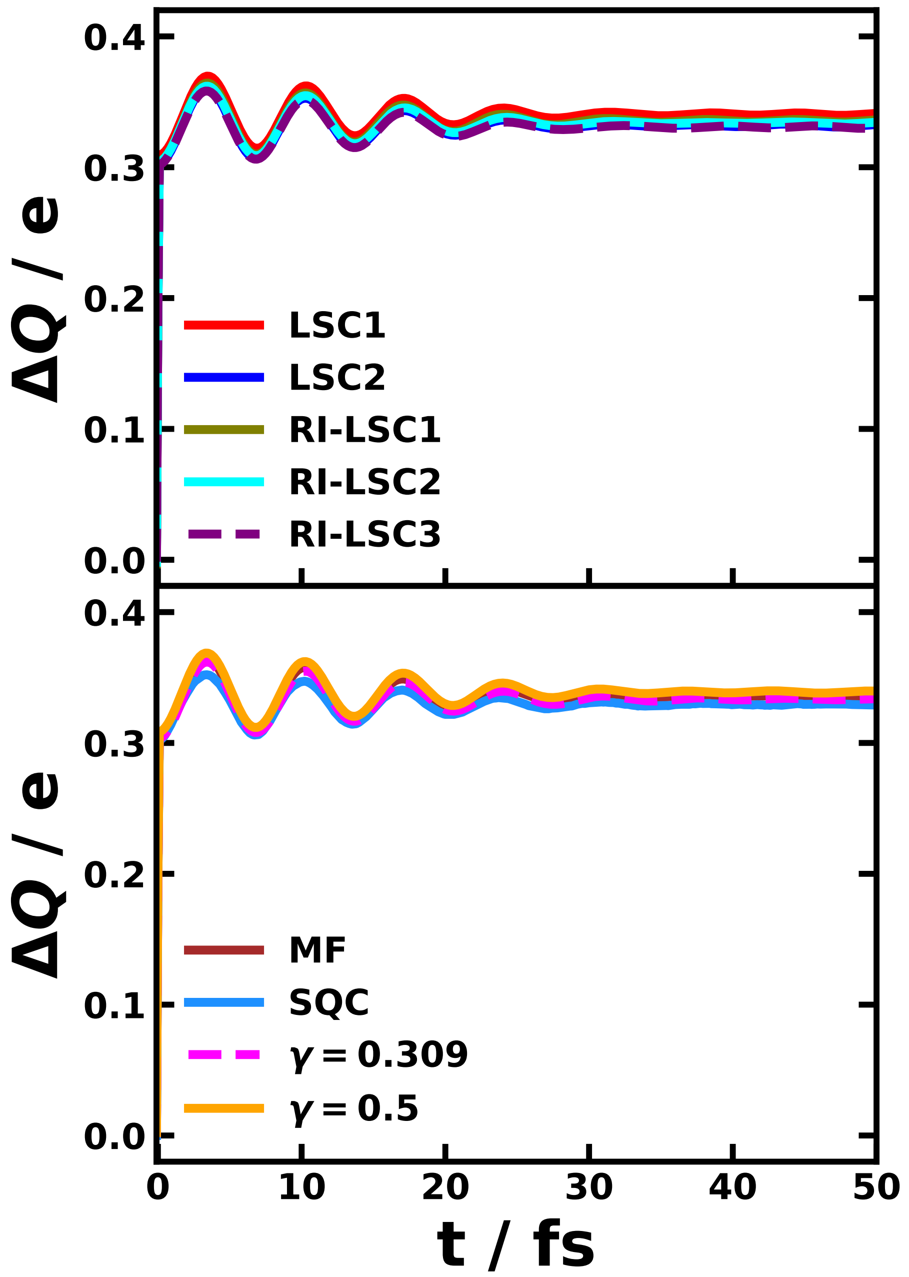

FIG. S2. Time-dependent charge transfer amount from the donor moiety to the acceptor moieties of Y6 via the non-perturbative approach obtained with different nonadiabatic dynamics. The time-dependent change transfer amount is calculated by $\Delta Q(t) = \sum_{j\neq g}^{F} \Delta Q_j \sigma_{jj}(t)$, where ΔQ_j is the state-dependent charge transfer amount as shown in Table S1.

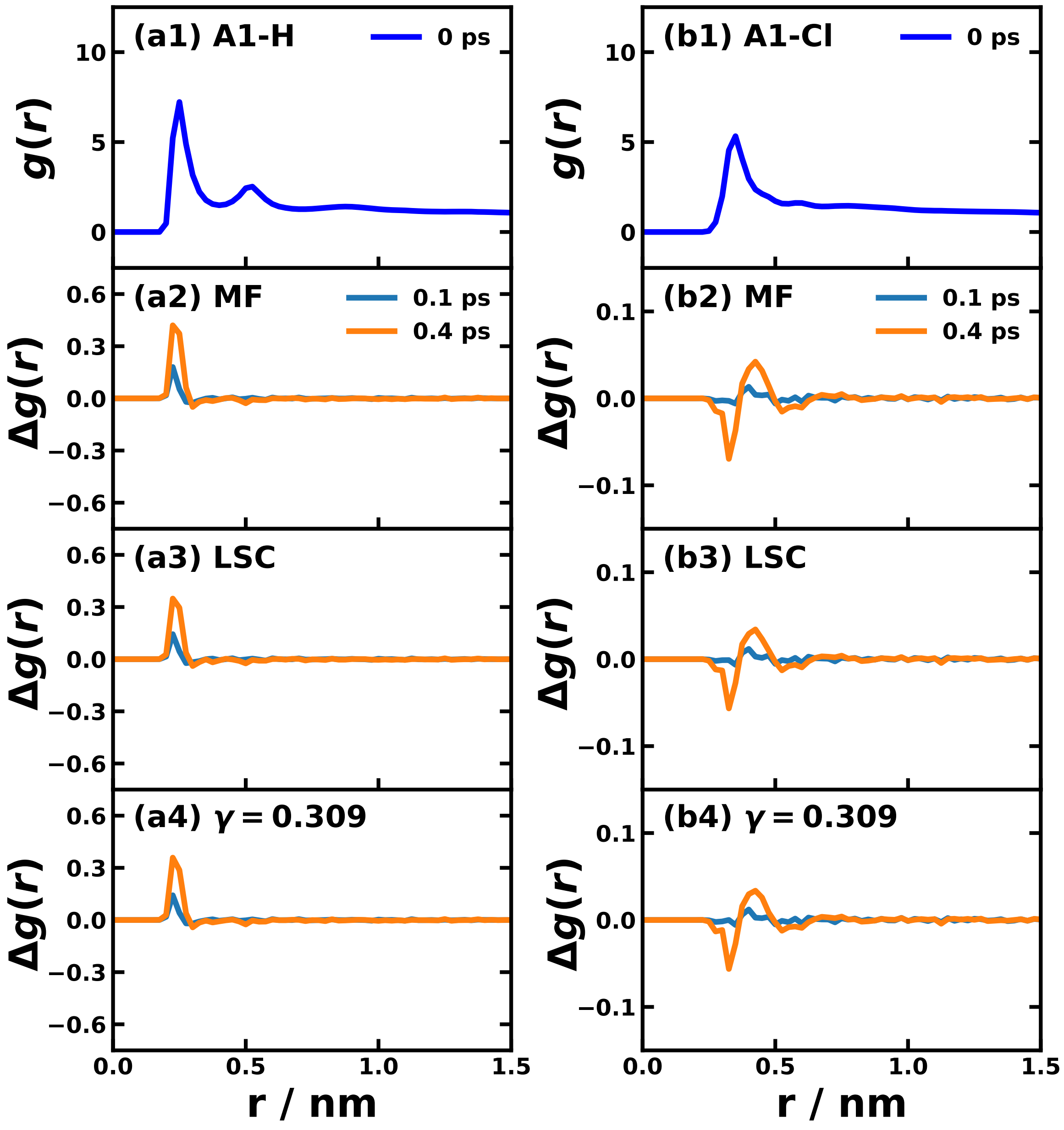

FIG. S3. Radial distribution function (RDF) of the hydrogen (left) and chlorine (right) atoms of chloroform with respect to the surface of acceptor moiety A1 of Y6. The first row (a1,b1) corresponds to the RDF of the equilibrium ground state, the rows 2 to 4 correspond to the RDF changes up to 0.1 ps (blue) and 0.4 ps (orange) after the applied light pulse in non-perturbative approach via MF (a2,b2), LSC (a3,b3), and CMM with $\gamma = 0.309$ (a4,b4).

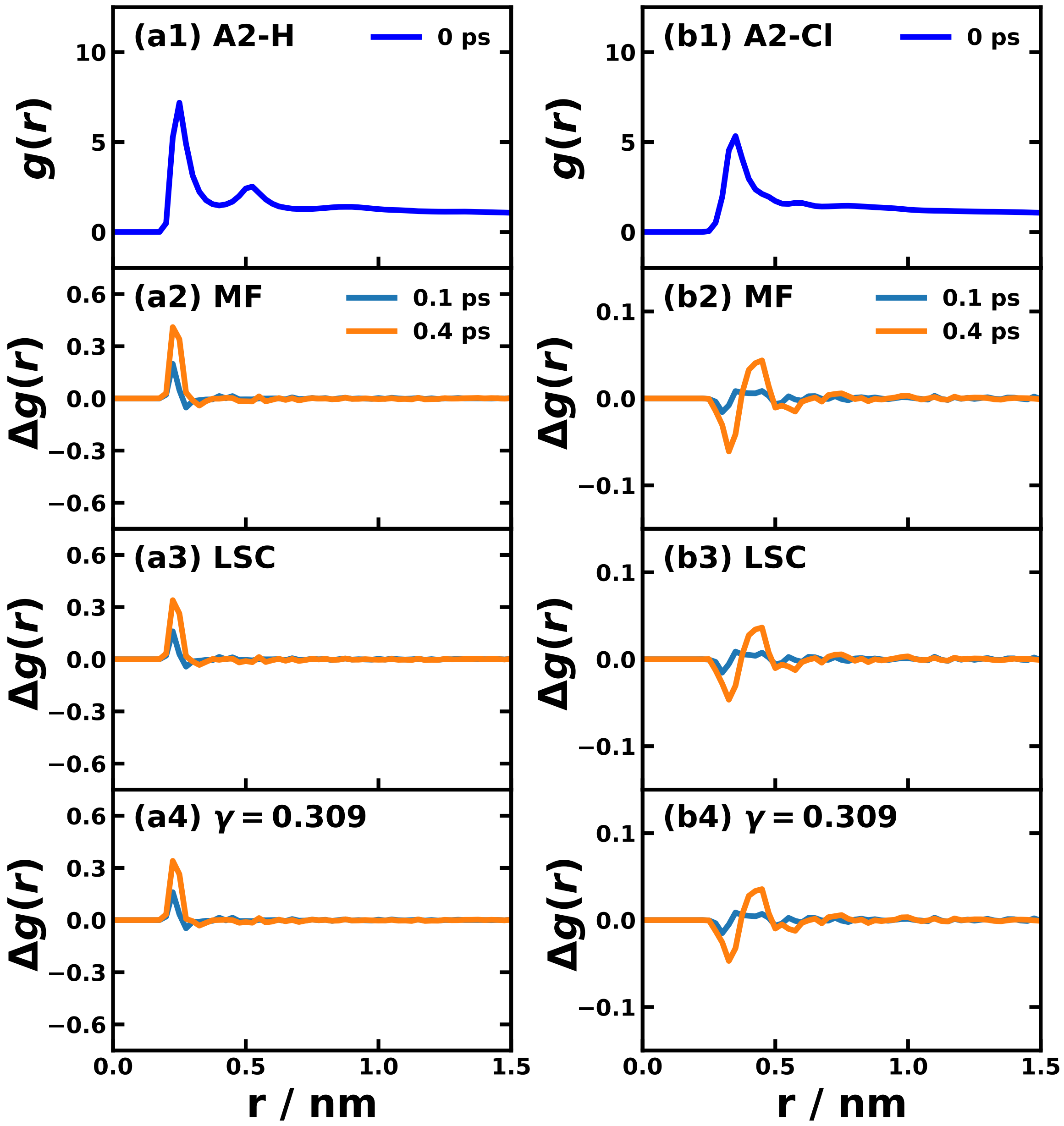

FIG. S4. Radial distribution function (RDF) of the hydrogen (left) and chlorine (right) atoms of chloroform with respect to the surface of acceptor moiety A2 of Y6. The first row (a1,b1) corresponds to the RDF of the equilibrium ground state, the rows 2 to 4 correspond to the RDF changes up to 0.1 ps (blue) and 0.4 ps (orange) after the applied light pulse in non-perturbative approach via MF (a2,b2), LSC (a3,b3), and CMM with $\gamma = 0.309$ (a4,b4).

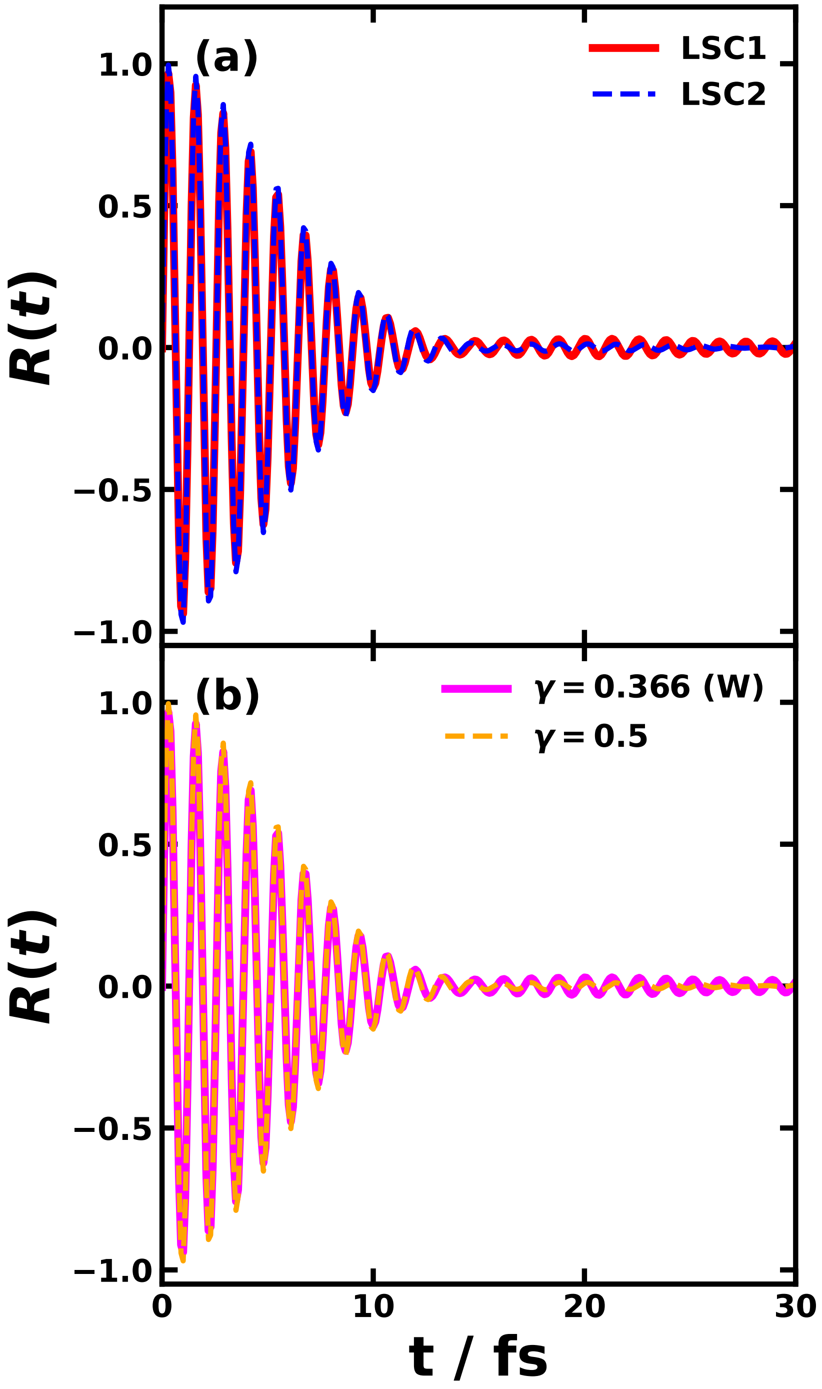

FIG. S5. Time-domain response functions of C153's benzene solution at 300 K calculated using the perturbative approach via nonadiabatic dynamics including LSC1 and LSC2 (upper panel) and CMM with ZPE parameters $\gamma = 0.366$ (SPM-W) and $\gamma = 0.5$ (lower panel). The results are normalized per their amplitude maxima.

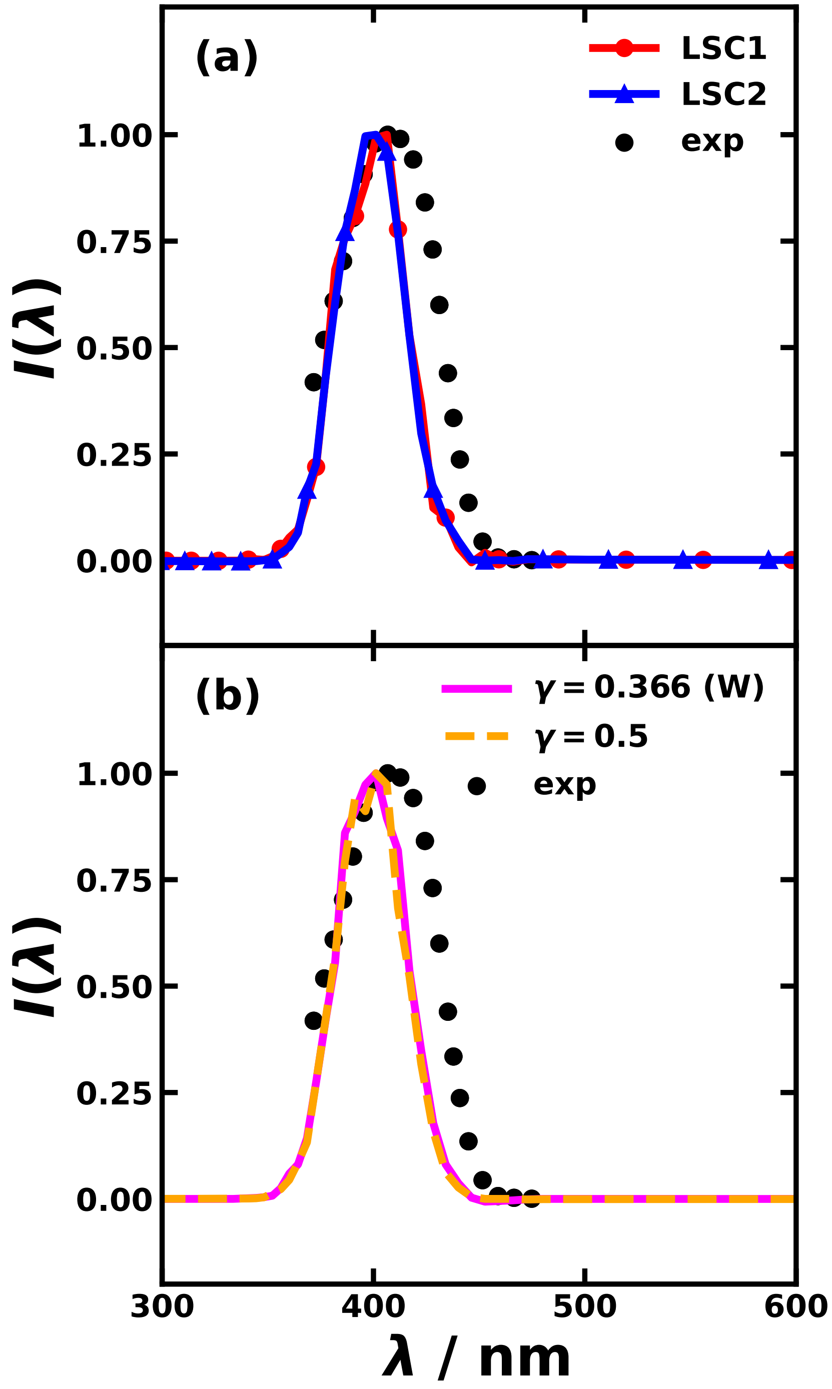

FIG. S6. Linear absorption spectra of C153's benzene solution at 300 K as a function of wavelength λ (nm) calculated using the perturbative approach via nonadiabatic dynamics including LSC1 and LSC2 (upper panel) and CMM with ZPE parameters $\gamma = 0.366$ (SPM-W) and $\gamma = 0.5$ (lower panel). The experiment results are adapted from Ref. 27. The simulated peak positions are not shifted and the spectral lineshapes are normalized according to the maximum peak amplitude.

REFERENCES

[1] Z. Hu and X. Sun, “All-Atom Nonadiabatic Semiclassical Mapping Dynamics for Photoinduced Charge Transfer of Organic Photovoltaic Molecules in Explicit Solvents,” J. Chem. Theory Comput. **18**, 5819–5836 (2022).

[2] H.-D. Meyer and W. H. Miller, “A Classical Analog for Electronic Degrees of Freedom in Nonadiabatic Collision Processes,” J. Chem. Phys. **70**, 3214–3223 (1979).

[3] G. Stock and M. Thoss, “Semiclassical Description of Nonadiabatic Quantum Dynamics,” Phys. Rev. Lett. **78**, 578–581 (1997).

[4] X. Gao, M. A. C. Saller, Y. Liu, A. Kelly, J. O. Richardson, and E. Geva, “Benchmarking Quasiclassical Mapping Hamiltonian Methods for Simulating Electronically Nonadiabatic Molecular Dynamics.” J. Chem. Theory Comput. **16**, 2883–2895 (2020).

[5] M. A. C. Saller, A. Kelly, and J. O. Richardson, “On the Identity of the Identity Operator in Nonadiabatic Linearized Semiclassical Dynamics,” J. Chem. Phys. **150**, 071101 (2019).

[6] M. A. C. Saller, A. Kelly, and J. O. Richardson, “Improved Population Operators for Multi-State Nonadiabatic Dynamics with the Mixed Quantum-Classical Mapping Approach,” Faraday Discuss. **221**, 150–167 (2020).

[7] X. Gao and E. Geva, “A Nonperturbative Methodology for Simulating Multidimensional Spectra of Multiexcitonic Molecular Systems via Quasiclassical Mapping Hamiltonian Methods.” J. Chem. Theory Comput. **16**, 6491–6502 (2020).

[8] X. Gao, Y. Lai, and E. Geva, “Simulating Absorption Spectra of Multiexcitonic Systems via Quasiclassical Mapping Hamiltonian Methods.” J. Chem. Theory Comput. **16**, 6465–6480 (2020).

[9] X. He and J. Liu, “A New Perspective for Nonadiabatic Dynamics with Phase Space Mapping Models,” J. Chem. Phys. **151**, 024105 (2019).

[10] X. He, Z. Gong, B. Wu, and J. Liu, “Negative Zero-Point-Energy Parameter in the Meyer-Miller Mapping Model for Nonadiabatic Dynamics,” J. Phys. Chem. Lett. **12**, 2496–2501 (2021).

[11] J. Liu, X. He, and B. Wu, “Unified Formulation of Phase Space Mapping Approaches for Nonadiabatic Quantum Dynamics,” Acc. Chem. Res. **54**, 4215–4228 (2021).

[12] X. He, B. Wu, Y. Shang, B. Li, X. Cheng, and J. Liu, “New Phase Space Formulations and Quantum Dynamics Approaches,” WIREs Comput. Mol. Sci. **12**, e1619 (2022).

[13] J. E. Runeson and J. O. Richardson, “Spin-Mapping Approach for Nonadiabatic Molecular Dynamics,” J. Chem. Phys. **151**, 044119 (2019).

[14] J. E. Runeson and J. O. Richardson, “Generalized Spin Mapping for Quantum-Classical Dynamics,” J. Chem. Phys. **152**, 084110 (2020).

[15] S. J. Cotton and W. H. Miller, “Symmetrical Windowing for Quantum States in Quasi-Classical Trajectory Simulations: Application to Electronically Non-Adiabatic Processes,” J. Chem. Phys. **139**, 234112 (2013).

[16] S. J. Cotton, K. Igumenshchev, and W. H. Miller, “Symmetrical Windowing for Quantum States in Quasi-Classical Trajectory Simulations: Application to Electron Transfer,” J. Chem. Phys. **141**, 084104 (2014).

[17] S. J. Cotton and W. H. Miller, “A New Symmetrical Quasi-Classical Model for Electronically Non-Adiabatic Processes: Application to the Case of Weak Non-Adiabatic Coupling,” J. Chem. Phys. **145**, 144108 (2016).

[18] P. Ehrenfest, “Comment on the Approximate Validity of Classical Mechanics within Quantum Mechanics,” Z. Phys. **45**, 455–457 (1927).

[19] A. D. McLachlan, “A Variational Solution of the Time-Dependent Schrodinger Equation,” Mol. Phys. **8**, 39–44 (1964).

[20] Y. Guo, G. Han, and Y. Yi, “The Intrinsic Role of the Fusion Mode and Electron-Deficient Core in Fused-Ring Electron Acceptors for Organic Photovoltaics,” Angew. Chem. Int. Ed. **61**, e202205975 (2022).

[21] E. Epifanovsky, A. T. B. Gilbert, X. Feng, J. Lee, Y. Mao, N. Mardirossian, P. Pokhilko, A. F. White, M. P. Coons, *et al.*, “Software for the Frontiers of Quantum Chemistry: An Overview of Developments in the Q-Chem 5 Package,” J. Chem. Phys. **155**, 084801 (2021).

[22] D. M. Chipman, “Charge Penetration in Dielectric Models of Solvation,” J. Chem. Phys. **106**, 10194–10206 (1997).

[23] B. H. Besler, K. M. Merz Jr., and P. A. Kollman, “Atomic Charges Derived from Semiempirical Methods,” J. Comput. Chem. **11**, 431–439 (1990).

[24] A. A. Voityuk and N. Rösch, “Fragment Charge Difference Method for Estimating Donor–Acceptor Electronic Coupling: Application to DNA π-Stacks,” J. Chem. Phys. **117**, 5607–5616 (2002).

[25] M. J. Frisch, G. W. Trucks, H. B. Schlegel, G. E. Scuseria, M. A. Robb, J. R. Cheeseman, G. Scalmani, V. Barone, G. A. Petersson, H. Nakatsuji, *et al.*, “Gaussian16 Revision C.01,” Gaussian Inc. Wallingford CT (2016).

[26] M. J. Frisch, G. W. Trucks, H. B. Schlegel, G. E. Scuseria, M. A. Robb, J. R. Cheeseman, G. Scalmani, V. Barone, G. A. Petersson, H. Nakatsuji, X. Li, M. Caricato, A. V. Marenich, J. Bloino, B. G. Janesko, R. Gomperts, B. Mennucci, H. P. Hratchian, J. V. Ortiz, A. F. Izmaylov, J. L. Sonnenberg, D. Williams-Young, F. Ding, F. Lipparini, F. Egidi, J. Goings, B. Peng, A. Petrone, T. Henderson, D. Ranasinghe, V. G. Zakrzewski, J. Gao, N. Rega, G. Zheng, W. Liang, M. Hada, M. Ehara, K. Toyota, R. Fukuda, J. Hasegawa, M. Ishida, T. Nakajima, Y. Honda, O. Kitao, H. Nakai, T. Vreven, K. Throssell, J. A. Montgomery, Jr., J. E. Peralta, F. Ogliaro, M. J. Bearpark, J. J. Heyd, E. N. Brothers, K. N. Kudin, V. N. Staroverov, T. A. Keith, R. Kobayashi, J. Normand, K. Raghavachari, A. P. Rendell, J. C. Burant, S. S. Iyengar, J. Tomasi, M. Cossi, J. M. Millam, M. Klene, C. Adamo, R. Cammi, J. W. Ochterski, R. L. Martin, K. Morokuma, O. Farkas, J. B. Foresman, and D. J. Fox, “Gaussian16 Revision C.01,” Gaussian Inc. Wallingford CT (2016).

[27] M. L. Horng, J. A. Gardecki, A. Papazyan, and M. Maroncelli, “Subpicosecond Measurements of Polar Solvation Dynamics: Coumarin 153 Revisited,” J. Phys. Chem. **99**, 17311–17337 (1995).
