# Supplementary material for: Direct All-Atom Nonadiabatic Semiclassical Simulations for Electronic Absorption Spectroscopy of Organic Photovoltaic Non-Fullerene Acceptor in Solution
Source: J Phys Chem Lett. 2025 Apr 25;16(18):4463–73. doi: 10.1021/acs.jpclett.5c00714 (PMC12067435; doi:10.1021/acs.jpclett.5c00714)
Supplement: Supplementary file 2 — jz5c00714_si_002.pdf [file jz5c00714_si_002.pdf]

jz-2025-00714a.R1

Name: Peer Review Information for "Direct All-Atom Nonadiabatic Semiclassical Simulations for Electronic Absorption Spectroscopy of Organic Photovoltaic Non-Fullerene Acceptor in Solution"

First Round of Reviewer Comments

Reviewer: 1

Comments to the Author

The manuscript of Liu and Sun demonstrates the applicability of several semiclassical methods combined with all-atomic electronic structure calculations to produce spectra of non-fullerene Y6 compound that is of interest to organic photovoltaic materials. The key message here is that the methods are capable of producing the spectral lineshapes without the need for additional tuning, as shown via direct comparison with the experimental spectrum. The authors also present a comparative analysis of a range of perturbative and non-perturbative semiclassical methods. Overall, the work is solid and well presented. The SI is particularly helpful for understanding the formulation of these methods and the differences between them. I believe that the authors have achieved what they have aimed for in this work, so I recommend the work for acceptance. However, I believe there is still a potential for an improvement that the authors can make in this work.

1) It is not very clear to me which of these methods is the best in this and potentially other situations – the results shown in Figs 2, 3, 5, and 6 look nearly the same for all the methods considered, even though the authors highlight some differences. Out of these differences, I can appreciate the negative peak given by SQC method in Figure 3, panel b2. However, I don't see how "SQC generates more oscillatory S1 population and weaker coherences than the other methods" in Figure 5.

2) It would be extremely helpful to consider systems/conditions that could highlight the differences in the performance of these various methods. Even if the methods still produce the results that are all consistent with each other, it would be good to demonstrate them working for a other molecules – not just for a single one. While one wishes to believe they would still work as good as in this case, we cannot take this for granted. Thus, more

examples would be desirable. I don't necessarily intend to nudge the authors into doing such calculations since they may be quite expensive and challenging, but if there is a possibility to extend the present example to include more systems, it would certainly help strengthen the work.

3) While seeing the results of the semiclassical calculations, I wondered how would one of the most popular methods – Tully's FSSH perform here. Potentially, it would show some deficiencies of that simple methodology and help highlight the strengths of the current methods. Can the authors provide such a comparison?

4) In the paragraph after Eq. 7, it probably should say "the second line of Eq. 7", not "Eq. 6"

5) In the SI, the paragraph after Eq. S1: E(t) = e E(t) cos(omega t – k r) – how is the k*r dependence handled in this work? It looks like one would need to do the integration over the electronic coordinate r including the "cos" term when computing the corresponding matrix elements. Or is there some other approximation used that would help to get rid of the r-dependent term?

Reviewer: 2

Comments to the Author

In this manuscript, the authors present a comparison of perturbative and nonpertubative approaches of field-matter interaction for simulating linear absorption spectra in the solvated Y6 system in which nonadiabatic effects are present using a semiclassical mapping procedure (MMTS Hamiltonian). The methods employed combine the essential physics necessary to model the spectrum, which is challenging to bring all together, showing good agreement with experiment. The science appears robust, but the clarity of the presentation of the methods and results could be improved. I am also unsure what physical insight is provided in this manuscript (I don't know that I learned anything about the Y6 system), so I question if JPCLett is the best choice and wonder if JCTC might be a better fit. Below are some specific suggestions for improving the manuscript.

1. In the paragraph describing the drawbacks of the cumulant method, there could be come clarification of the wording, "It effectively assumes Gaussian distribution of the

energy gap..., thereby invoking harmonic approximation." This statement could be more precise, e.g. "Truncation at second order in the expansion is exact for a Gaussian distribution of the energy gap..., which is true for harmonic potentials of identical curvature." Also, the statement "Thus, a main drawback of the second-order cumulant approach is that it cannot describe dynamical effects on anharmonic PES,..." is not completely accurate, as these effects can be partially described through sampling on an anharmonic PES, see work by Markland and co-workers, Ref. 32.

2. In the next paragraph, the states included in the Hamiltonian, S1, S2, and S6 are described as "the most significant,"and later it is mentioned that these states have large oscillator strengths from the ground state. It makes sense that these would be the initially excited states, but it seems that coupling to dark states S3, S4, and S5 might be relevant, and information could also be included about these states in the SI. Although the information about the electronic structure details are given the SI, some details should be given in the main manuscript. I realise that the authors are trying to save space and keeping the manuscript letter length, but it would be useful if some description of the electronic structure method and how environmental sampling was done was included, including that the dynamics was done with a force field, but I think the only way the excited state is modelled is via the excited state charges? This is key to making sure the reader understands how vibronic effects, anharmonic effects, and environmental effects are included in the simulation, as only the explanation of the nonadiabatic dynamics is given.

3. Equation 6 does not have theta(t) defined.

4. The field details are not given for the results in Figure 2. There is mention of one time step, but no other information. If the goal is to show the effect of the field in the non-perturbative simulations, why not include the field for a longer period of time?

5. How are the ZPE parameters chosen? The RI-LSC1-3 methods are not defined in the main manuscript.

6. The main result seems to be that the mapping methods and the perturbative and non-perturbative approaches all give the similar spectra and RDF plots, but I don't know that this point is clearly set up in the motivation. For a methods comparison paper, maybe JCTC is more appropriate. It would be useful for the authors to emphasize the novelty of their work.

7. It also isn't clear that nonadiabatic effects are important in this absorption spectrum. There is population oscillation between states 2 and 6, but how is the coupling between the states determined and what does this oscillation frequency correspond to? Is

this a vibronic coherence? What would the spectrum look like if only the vibronic effects were included for each transition and there were no nonadiabatic effects considered?

8. The labels for the reduced density matrix dynamics were confusing – e.g. ground and 1st excited state have a sigma_14 label.

Author's Response to Peer Review Comments:

Prof. Dr. Xiang Sun
Division of Arts and Sciences
New York University Shanghai
567 West Yangsi Road
Shanghai, 200124, China
Tel: (+86) 21-20596163
E-mail: xiang.sun@nyu.edu

April 2, 2025

Senior Editor, *Journal of Physical Chemistry Letters*
College of Chemistry, Key Laboratory of Theoretical and Computational Photochemistry of Ministry of Education
Beijing Normal University
Beijing 100875, China

Dear Editor:

We are very grateful to the reviewers for their thoughtful comments on our manuscript titled “Direct All-Atom Nonadiabatic Semiclassical Simulations for Electronic Absorption Spectroscopy of Organic Photovoltaic Non-Fullerene Acceptor in Solution", by Zengkui Liu and Xiang Sun. We revised the manuscript to address the reviewers’ comments as detailed below.

Reviewer #1
Recommendation: This paper is probably publishable, but major revision is needed; I do not need to see future revisions.

Reviewer’s comments: “The manuscript of Liu and Sun demonstrates the applicability of several semiclassical methods combined with all-atomic electronic structure calculations to produce spectra of non-fullerene Y6 compound that is of interest to organic photovoltaic

materials. The key message here is that the methods are capable of producing the spectral lineshapes without the need for additional tuning, as shown via direct comparison with the experimental spectrum. The authors also present a comparative analysis of a range of perturbative and non-perturbative semiclassical methods. Overall, the work is solid and well presented. The SI is particularly helpful for understanding the formulation of these methods and the differences between them. I believe that the authors have achieved what they have aimed for in this work, so I recommend the work for acceptance. However, I believe there is still a potential for an improvement that the authors can make in this work."

Authors' reply: We appreciate Reviewer #1's acknowledgment and comments on this work. We will address the questions point-by-point below.

Questions/critical comments:

1. Reviewer's comment: "It is not very clear to me which of these methods is the best in this and potentially other situations – the results shown in Figs 2, 3, 5, and 6 look nearly the same for all the methods considered, even though the authors highlight some differences. Out of these differences, I can appreciate the negative peak given by SQC method in Figure 3, panel b2. However, I don't see how 'SQC generates more oscillatory S1 population and weaker coherences than the other methods' in Figure 5."

   Authors' reply: Thank you for raising this question! In the current case, all the semiclassical dynamics tested here except for SQC method should be fine in the simulation of linear spectra of Y6 solution. The reason for most of semiclassical dynamics giving rise to similar spectra lies in the fact that the system is at a high temperature 300 K and the maximum reorganization energy is small, around 20 kJ/mol (see Table S1). Therefore, most of the semiclassical dynamics are unsurprisingly able to capture the nonadiabatic dynamics in the Y6 system, which reflects that the system falls into the "easy" parameter region, i.e., high temperature and small reorganization energy, for nonadiabatic dynamics as shown in the recent benchmark work (Ref. 68). Additionally, the SQC shows more oscillatory S1 population dynamics can be seen in Fig. 5's σ_{11} panel and Fig. S1(g) panel; the SQC shows weaker coherences than other methods can be seen in Fig. 5's offdiagonal panels where the blue curves have a smaller amplitude than other nonadiabatic methods.

   To this end, we have added the references to the corresponding Figures in the main text on Page 17 as below: "the SQC generates more oscillatory S_1 population and weaker coherences than the other methods [see Fig. 5 and Fig. S1(g)]."

2. Reviewer's comment: "It would be extremely helpful to consider systems/conditions that could highlight the differences in the performance of these various methods. Even if the methods still produce the results that are all consistent with each other, it would be good to demonstrate them working for a other molecules – not just for a single one. While one wishes to believe they would still work as good as in this case, we cannot take this for granted. Thus, more examples would be desirable. I don't necessarily intend to nudge the authors into doing such calculations since they may be quite expensive and challenging, but if there is a possibility to extend the present example to include more systems, it would certainly help strengthen the work."

   Authors' reply: Thanks for your suggestion. We tested the perturbative approach to the Coumarin 153 (C153) benzene solution and now we included the corresponding simulation result in the Supporting Information. The model for C153 involves only the ground (S_0) state and the S_1 excited state. As shown in Figures S5 and S6, the simulated spectra reproduce the experimental peak position and about 60% peak width for the S_1-state absorption of the C153 in benzene solution. The simulated spectra obtained with different semiclassical methods are rather similar, which suggests that C153 benzene solution at 300 K, similar to the Y6 case, also falls into the parameter space that is friendly for various semiclassical dynamics, as shown in recent benchmark work Ref. 68.

   To this end, we added the following text on Page 14 to briefly mention the newly added C153 system: "We also test the perturbative approach on coumarin 153 (C153)'s benzene solution at 300 K. The ground S_0 state and the first excited S_1 state of C153 are considered here. The response function and the linear spectra are shown in Figs. S5 and S6 in the Supporting Information. The simulated spectra reproduce the peak position of the experimental result and about 60% peak width for the absorption of $S_0 \rightarrow S_1$ transition. Additionally, the simulated spectra of the C153 solution obtained with various semiclassical dynamics methods are very similar, also seen in the Y6 case. "

3. Reviewer's comment: "While seeing the results of the semiclassical calculations, I wondered how would one of the most popular methods – Tully's FSSH perform here. Potentially, it would show some deficiencies of that simple methodology and help highlight the strengths of the current methods. Can the authors provide such a comparison?"

   Authors' reply: Thank you for raising this question. It is natural to wonder whether widely adopted FSSH would perform in spectroscopy. However, we decided to not include FSSH method in this work since the current perturbative treatment for the response function requires coherence-tocoherence time correlation function (TCF)

and the non-perturbative treatment requires population-to-coherence TCF, which are not well-defined in FSSH. It might be possible with more advanced surface hopping methods with decoherence, and they are beyond the scope of this work.

4. Reviewer's comment: " In the paragraph after Eq. 7, it probably should say 'the second line of Eq. 7', not 'Eq. 6'. "

   Authors' reply: Thank you for pointing it out. This has been corrected.

5. Reviewer's comment: "In the SI, the paragraph after Eq. S1: $\mathbf{E}(t)$ = $\mathbf{e}\hat{}E(t)\cos(\omega t\breve{}\mathbf{k}\cdot\mathbf{r})$ — how is the $\mathbf{k}\cdot\mathbf{r}$ dependence handled in this work? It looks like one would need to do the integration over the electronic coordinate $\mathbf{r}$ including the 'cos' term when computing the corresponding matrix elements. Or is there some other approximation used that would help to get rid of the $\mathbf{r}$-dependent term?"

   Authors' reply: Thank you for the reminder. We forgot to mention that we evoked the long wavelength approximation, which assumes the molecular size is much smaller than the wavelength of the incident field and thus the electric field around the Y6 molecule can be considered as uniform. Under this approximation, the dot product $\mathbf{k} \cdot \mathbf{r}$ (here, $\mathbf{r}$ is 3D spatial coordinate, not the electronic coordinate) is treated as a constant so it can be neglected in the simulation.

   To this end, we added the following sentence in SI to clarify the approximation: "Here, we invoke the long-wavelength approximation, which assumes the molecular size is much smaller than the wavelength of the incident field, thus the electric field at different positions and $\mathbf{k} \cdot \mathbf{r}$ can be treated as constants."

Reviewer #2

Recommendation: While the work is good and publishable, a more appropriate journal is recommended such as JCTC.

Reviewer's comment: "In this manuscript, the authors present a comparison of perturbative and nonpertubative approaches of field-matter interaction for simulating linear absorption spectra in the solvated Y6 system in which nonadiabatic effects are present using a semiclassical mapping procedure (MMTS Hamiltonian). The methods employed combine the essential physics necessary to model the spectrum, which is challenging to bring all together, showing good agreement with experiment. The science appears robust, but the clarity of the presentation of the methods and results could be improved. I am also unsure what physical insight is provided in this manuscript (I don't know that I learned anything about the Y6 system), so I question if JPCLett is the best choice and wonder if JCTC might be a better fit. Below are some specific suggestions for improving the manuscript."

Authors' reply: We thank the Reviewer #2 for the acknowledgment and comments. The novelty of this work includes: (1) proposing the simulation protocol for linear spectroscopy using a consistent description of all-atom multistate anharmonic Hamiltonian, nonadiabatic semiclassical dynamics, and perturbative and non-perturbative spectroscopic treatment, and demonstrating in Y6 chloroform solution at room temperature; (2) the dynamical information obtained from the direct simulation for Y6 system, such as the time-dependent radial distribution function that shows how solvent reorganizes in response to the photoinduced charge transfer as well as the population and coherence dynamics of multiple electronic states in the Y6 system along with the time-dependent charged transferred amount. We believe both the direct simulation protocol and the observations of Y6 after explicit light-matter interaction provide new physical insights to better understand the microscopic events in the spectroscopic measurement of the Y6 solution. Thus, this work perfectly suits the scope of *Journal of Physical Chemistry Letters (JPCL)*, which is a prestigious journal that "*rapidly disseminates high-quality communications at the forefront of physical chemistry*".

Questions/critical comments:

1. Reviewer's comment: "In the paragraph describing the drawbacks of the cumulant method, there could be come clarification of the wording, 'It effectively assumes Gaussian distribution of the energy gap ..., thereby invoking harmonic approximation.' This statement could be more precise, e.g. 'Truncation at second order in the expansion is exact for a Gaussian distribution of the energy gap ..., which is true for harmonic potentials of identical curvature.' Also, the statement 'Thus, a main

drawback of the second-order cumulant approach is that it cannot describe dynamical effects on anharmonic PES,…' is not completely accurate, as these effects can be partially described through sampling on an anharmonic PES, see work by Markland and co-workers, Ref. 32."

Authors' reply: Thank you for the comments. It is a very good point and many readers might be confused about PESs, since in this context, there are two PES concepts, one is the sampling PES, and the other is the PES that is used to propagate dynamics. The core of the problem is whether sampling on anharmonic PES to get energy-gap TCF and subsequently constructing shifted harmonic models would include any anharmonic dynamical effects. We distinguish the anharmonic effects from sampling and dynamics. Here, the first anharmonic effect from sampling is widely used in previous work as you mentioned (Ref. 32), and also our recently proposed general multi-state harmonic model Hamiltonian construction (Ref. 42). Due to the central limit theorem, Gaussian approximation to the energy gap might be a good one for systems with large degrees of freedom, and we have seen many successful applications of BOM in reproducing the spectroscopic signature. However, when one uses the energy-gap TCF to map to a shifted harmonic model (or BOM), the effective PESs become harmonic, and then performing quantum dynamics on the harmonic PESs won't capture any dynamical anharmonic effect, which is what we meant by "dynamical effects on anharmonic PES". To test for the dynamical anharmonic effect, one would have to compare the full quantum dynamics obtained with the realistic anharmonic Hamiltonian and the corresponding effective harmonic model Hamiltonian, which can be quite challenging and out of the scope of this work.

To this end, to make this point clearer, we revised the following sentences on the introduction: "In other words, if a system is accurately described by shifted harmonic potentials of identical curvature, then the Gaussian distribution of energy gap and hence the second order cumulant expansion would be exact, but for realistic systems, the second order cumulant method is an approximation. …It should be distinguished between the anharmonic PES effects from sampling and dynamical perspectives. Sampling on anharmonic PES is widely adopted when obtaining energy-gap TCFs, but it does not fully capture the anharmonic effect in the dynamics of spectroscopic response. It is noted that whether or not to construct BOM explicitly, using spectral density in the second-order cumulant approach invokes the harmonic approximation to the effective PES that will be used subsequently for dynamics, which is known analytically. "

2. Reviewer's comment: "In the next paragraph, the states included in the Hamiltonian, S1, S2, and S6 are described as 'the most significant,' and later it is mentioned that

these states have large oscillator strengths from the ground state. It makes sense that these would be the initially excited states, but it seems that coupling to dark states S3, S4, and S5 might be relevant, and information could also be included about these states in the SI. Although the information about the electronic structure details are given the SI, some details should be given in the main manuscript. I realise that the authors are trying to save space and keeping the manuscript letter length, but it would be useful if some description of the electronic structure method and how environmental sampling was done was included, including that the dynamics was done with a force field, but I think the only way the excited state is modelled is via the excited state charges? This is key to making sure the reader understands how vibronic effects, anharmonic effects, and environmental effects are included in the simulation, as only the explanation of the nonadiabatic dynamics is given."

Authors' reply: Thank you for raising the great questions. The selection of excited states S1, S2, and S6 is for two reasons. First, these bright excited states have a large oscillator strength and excitation energies lower than 3.0 eV, which will contribute most significantly to the linear absorption spectroscopy and it can be corroborated by the direct comparison with the three major peaks in the experimental spectra. Second, neglecting the dark states such as S3, S4, S5 can be rationalized by the dominant ultrafast time scale for the absorption response function is 20 fs, during which the relaxation from initially excited bright states to the dark states is expected to be not significant. Plus, it is not thermodynamically favorable to have population transfer from the most populated state S1 to other higher excited dark states in such a short time scale. We added more details of the dark states in the Supporting Information, although we don't include them in the nonadiabatic dynamics calculation. For the excitedstate force fields, we indeed used the atomic charges corresponding to each of the bright excited-state electron distributions, which was described in the Supporting Information.

Following your suggestion, we added the simulation summary section to the end of the main text of the manuscript: " The excitation energies, transition dipoles from the ground state to excited states, diabatic couplings, and atomic charges were calculated with time-dependent density functional theory (TDDFT) on the level of ω*B97X-D/6-31G(d,p) using polarizable continuum model of $\epsilon_0 = 3.0$ and the tuned range separation parameter is $\omega = 0.11$ using Q-Chem 6.0. The excited states (S_1, S_2, S_6) were selected with the minimal oscillator strength threshold of 0.2 and maximum excitation energy threshold of 3.0 eV. The all-atom PESs are based on the generalized Amber force field, where the atomic charges and the excitation energies correspond to the TDDFT calculation for the ground state and the three excited states. The initial nuclear positions and momenta are sampled from equilibrated ground state Y6 with

1632 explicit chloroform solvent molecules in box $60.9 \times 60.9 \times 60.9$ ^{3} with periodic boundary conditions at 300 K. The nonadiabatic dynamics simulations of the Y6 molecule dissolved in the explicit solvent environment for the linear absorption spectra are performed using a nuclear time step of 0.1 fs and an electronic time step of 0.005 fs by averaging over 2×10^4 trajectories in the perturbative approach and 10^5 trajectories in the non-perturbative approach. For more details of the simulation, refer to the Supporting Information. "

At the end of the section quantum chemistry calculation of Y6 in the SI, we added the following sentences summarizing the properties of dark S3, S4, and S5 states: "We neglected the dark states in the nonadiabatic dynamical simulation since they are not expected to be heavily populated in the first tens of fs after the photoexcitation. For completeness, the excitation energies of the dark excited states S_3, S_4, and S_5 are 2.40, 2.46, and 2.50 eV, respectively and the oscillator strengths are 0.09, 0.02, and 0.03, respectively."

3. Reviewer's comment: "Equation 6 does not have theta(t) defined."

   Authors' reply: Thank you for pointing it out. Now we have added the definition of the Heaviside function $\theta(t)$ after Eq. 6.

4. Reviewer's comment: "The field details are not given for the results in Figure 2. There is mention of one time step, but no other information. If the goal is to show the effect of the field in the non-perturbative simulations, why not include the field for a longer period of time?"

   Authors' reply: Thanks for the question. The field details were added to the text on Page 11: "In the non-perturbative approach, the applied square laser pulse of strength $E = 2.57 \times 10^{10}$ V/m lasts for 0.1 fs.". Also, the caption of Fig. 2 was added: "the time-dependent polarization after the external laser pulse of strength $E = 2.57 \times 10^{10}$ V/m and duration 0.1 fs calculated with the non-perturbative approach". The motivation to use a short laser pulse is to make the nonequilibrium polarization close to the optical response function in the perturbative approach, since the polarization is the convolution between the response function and the external field. The response function contains the material-intrinsic information, and this is what we would like to know. On the other hand, we tested longer exposure time, and it basically generates similar linear absorption spectra, so we didn't include other exposure durations here.

5. Reviewer's comment: "How are the ZPE parameters chosen? The RI-LSC1-3 methods are not defined in the main manuscript."

Authors’ reply: Thank you for the comment. We added the definitions for the RI-LSC1–3 methods on Page 10: “The resolution of identity (RI) for the electronic DOF could improve the estimation of population, and combining the RI trick with the LSC approaches yields resolution-ofidentity linearized semiclassical 1–3 (RI-LSC1–3) methods (see Supporting Information for details).” ZPE choice is detailed in Supporting Information and the previous experience for making those choices for various semiclassical mapping dynamics could be found at Refs. 43 and 68 and the references therein.

6. Reviewer’s comment: “The main result seems to be that the mapping methods and the perturbative and non-perturbative approaches all give similar spectra and RDF plots, but I don’t know that this point is clearly set up in the motivation. For a methods comparison paper, maybe JCTC is more appropriate. It would be useful for the authors to emphasize the novelty of their work."

   Authors’ reply: Thank you for the comment. First and foremost, this is a method development paper and the novelty is to propose a computational protocol to simulate the absorption spectroscopy of liquid solution within a consistent treatment for anharmonic Hamiltonian, nonadiabatic dynamics and both perturbative and non-perturbative spectroscopic observables. The direct atomistic simulation approaches enable us to also have the insight regarding the electronic dynamics such as the population, coherence, and CT amount dynamics as well as the nuclear dynamics such as the RDFs during the spectroscopic event. We tested this protocol using different semiclassical mapping dynamics and saw that most of the methods yield similar spectroscopy, indicating that this Y6 liquid solution at room temperature can be captured well with these methods, which is not the only discovery of this work but a useful observation for future applications. So this work is beyond simple methods comparison since the entire atomistic nonadiabatic dynamical protocol for direct simulation of linear spectra was not introduced before.

   The novelty of the work was mentioned in the introduction on Page 5: “In this work, we present a direct dynamical protocol for simulating the linear electronic spectroscopy of molecules in liquid solution with atomistic details, which has a consistent theoretical treatment for several aspects, including anharmonic PES, environmental effect, vibronic effect, finite temperature effect, as well as nonadiabatic dynamics that could allow population transfer and coherences between multiple electronically excited states, plus a realistic simulation of the field-matter interaction in both the perturbative and the non-perturbative approaches. Additionally, various nonadiabatic semiclassical mapping dynamics are also tested in the spectroscopic simulation.”

Together with the reply to your first comment where we summarize the novelty points, we believe JPCL is a suitable journal to publish this work.

7. Reviewer's comment: "It also isn't clear that nonadiabatic effects are important in this absorption spectrum. There is population oscillation between states 2 and 6, but how is the coupling between the states determined and what does this oscillation frequency correspond to? Is this a vibronic coherence? What would the spectrum look like if only the vibronic effects were included for each transition and there were no nonadiabatic effects considered?"

   Authors' reply: Thank you for raising this interesting question. We tested the nonadiabatic effect by disabling the electronic couplings between excited states, and the resulting preliminary linear spectra shows a difference from the originally simulated spectra. In particular, the S2 peak is even lower and S6 peak is even higher, which is more different from the experiment result than the simulated spectra with nonadiabatic effect, thus it is important to enable nonadiabatic dynamics in the spectroscopic simulation. The oscillation in the S2 and S6 population correlates well with the S2 and S6 coherence and they are simulated with the electronicvibrational coupling (or vibronic coupling), so we would say in the most general way that these are related to vibronic effect. However, to pinpoint the origin of such population/coherence oscillations, one probably needs to use two-dimensional electronic-vibrational spectroscopy, which is beyond this work. In addition, the electronic coupling between different electronic states was computed using the fragment charge difference (FCD) approach as mentioned in the SI.

   We added a sentence to highlight the importance of the nonadiabatic effect on Page 14: "Additionally, the nonadiabatic effects can be tested by turning off the electronic coupling between excited states but still having the vibronic coupling between electronic and nuclear DOF. The simulation without nonadiabatic effects yields a lower S2 peak and a higher S6 peak, which is more distinct from the experimental results than the original simulated spectra with nonadiabatic effects. This highlights the importance of the nonadiabatic effects in calculating the absorption spectroscopy. "

8. Reviewer's comment: "The labels for the reduced density matrix dynamics were confusing – e.g. ground and 1st excited state have a sigma_14 label."

   Authors' reply: Thanks for pointing it out. The reason we chose to use the last state as the ground state is to have consistency with our previous work. Besides defining the 1–4 labels to be excited S1, S2, S6, and the ground state, respectively in the caption, we modified Fig. 5 to reflect real and imaginary parts of σ_{14} to make this clearer.

Sincerely yours,

Xiang Sun

jz-2025-00714a.R2

Name: Peer Review Information for "Direct All-Atom Nonadiabatic Semiclassical Simulations for Electronic Absorption Spectroscopy of Organic Photovoltaic Non-Fullerene Acceptor in Solution"

Second Round of Reviewer Comments

Reviewer: 2

Comments to the Author

The authors emphasize that the Y6 system falls into the easy parameter region, therefore the various semiclassical dynamics lead to similar spectra. It would helpful if they could add information –Besides being at 300K and having a small reorganization energy, is there anything particular that can be clarified here regarding the easy parameter regime, e.g. mixing of states, etc.? It would be especially helpful if there were results for another system that was outside of this easy regime. I appreciate that the authors added results for C153 to the SI – but this system is also consider the easy regime and it isn't clear if it is necessary a priori to use nonadiabatic dynamics for the S0 and S1 states of this system – is there some criteria to use to judge when a nonadiabatic approach like the ones used here are necessary?

The response detailing why surface hopping was not included in this study because of the poorly defined TCFs helps to clarify differences in methods and would be good to add to the main manuscript (perhaps in the introduction?).

Author's Response to Peer Review Comments:

Prof. Dr. Xiang Sun

Division of Arts and Sciences

New York University Shanghai
567 West Yangsi Road

Shanghai, 200124, China

Tel: (+86) 21-20596163

E-mail: xiang.sun@nyu.edu

April 15, 2025

Senior Editor, *Journal of Physical Chemistry Letters*
College of Chemistry, Key Laboratory of Theoretical and Computational Photochemistry of Ministry of Education

Beijing Normal University

Beijing 100875, China

Dear Editor:

We are very grateful to the reviewers for their thoughtful comments on our manuscript titled "Direct All-Atom Nonadiabatic Semiclassical Simulations for Electronic Absorption Spectroscopy of Organic Photovoltaic Non-Fullerene Acceptor in Solution", by Zengkui Liu and Xiang Sun. We revised the manuscript to address the reviewers' comments as detailed below.

Reviewer #2
Recommendation: This paper is publishable subject to minor revisions noted.

Further review is not needed.

Reviewer's comments: "The authors emphasize that the Y6 system falls into the easy parameter region, therefore the various semiclassical dynamics lead to similar spectra. It would helpful if they could add information –Besides being at 300K and having a small reorganization energy, is there anything particular that can be clarified here regarding the easy parameter regime, e.g. mixing of states, etc.? It would be especially helpful if there were results for another system that was outside of this easy regime. I appreciate that the

authors added results for C153 to the SI – but this system is also consider the easy regime and it isn't clear if it is necessary a priori to use nonadiabatic dynamics for the S0 and S1 states of this system – is there some criteria to use to judge when a nonadiabatic approach like the ones used here are necessary?"

Authors' reply: We thank the reviewer for this insightful comment. Indeed, both the Y6 and C153 systems fall within what we refer to as the "easy parameter regime," where different semiclassical nonadiabatic dynamics methods tend to converge and often agree well with numerically exact results (see our benchmark study in J. Chem. Phys. 161, 024102 (2024)).

By "easy parameter regime", we broadly refer to systems where key factors— such as high temperature and small reorganization energy—collectively reduce the difference between nonadiabatic dynamical methods, leading to consistent predictions across methods. However, these factors are indicative rather than definitive.

To clarify this point, we have added the following sentence on Page 20:

"However, high temperature and small reorganization energy alone are not sufficient to guarantee agreement among various nonadiabatic dynamics methods. A benchmark comparison with exact results using reduced effective models—such as the multi-state harmonic (MSH) model [J. Chem. Phys. 155, 124105 (2021)]—is ultimately necessary, especially when all-atom quantum dynamics are not feasible."

As an example of a more complex case, we mention the carotenoid–porphyrin–C_{60} triad in tetrahydrofuran solvent, where different methods yield significantly divergent results [J. Chem. Theory Comp. 10, 5819-5836 (2022)]. While direct simulations of such a system are beyond the scope of the present work, we appreciate the reviewer's suggestion and acknowledge the value of future studies in more challenging regimes.

Reviewer's comments: "The response detailing why surface hopping was not included in this study because of the poorly defined TCFs helps to clarify differences in methods and would be good to add to the main manuscript (perhaps in the introduction?)."

Authors' reply: We thank the reviewer for this helpful suggestion. We have revised the Introduction to better clarify the reasoning behind our choice of nonadiabatic dynamics methods, particularly regarding the role of well-defined coherence in spectroscopic simulations. The updated sentence on Page 6 now reads:

"Additionally, various nonadiabatic semiclassical mapping dynamics are tested in the spectroscopic simulations. In both perturbative and non-perturbative treatments, the required TCFs involving electronic coherences are naturally defined. To ensure

consistency in treating these coherences, we include the mean-field Ehrenfest dynamics, while the fewest switches surface hopping is not considered, as defining such coherence-related TCFs within that framework is not straightforward."

We hope this revision improves clarity while maintaining a balanced presentation of different approaches.

Sincerely yours,

Xiang Sun
